# Proinflammatory Cytokine IL-6 and JAK-STAT Signaling Pathway in Myeloproliferative Neoplasms

**DOI:** 10.1155/2015/453020

**Published:** 2015-09-29

**Authors:** Vladan P. Čokić, Olivera Mitrović-Ajtić, Bojana B. Beleslin-Čokić, Dragana Marković, Marijana Buač, Miloš Diklić, Nada Kraguljac-Kurtović, Svetozar Damjanović, Pavle Milenković, Mirjana Gotić, Puri K. Raj

**Affiliations:** ^1^Institute for Medical Research, University of Belgrade, 11129 Belgrade, Serbia; ^2^Clinic of Endocrinology, Diabetes and Diseases of Metabolism, Clinical Center of Serbia, 11000 Belgrade, Serbia; ^3^Clinic of Hematology, Clinical Center of Serbia, 11000 Belgrade, Serbia; ^4^Medical Faculty, University of Belgrade, 11000 Belgrade, Serbia; ^5^Division of Cellular and Gene Therapies, Center for Biologics Evaluation and Research, Food and Drug Administration, Silver Spring, MD 20993, USA

## Abstract

The recent JAK1/2 inhibitor trial in myeloproliferative neoplasms (MPNs) showed that reducing inflammation can be more beneficial than targeting gene mutants. We evaluated the proinflammatory IL-6 cytokine and JAK-STAT signaling pathway related genes in circulating CD34^+^ cells of MPNs. Regarding laboratory data, leukocytosis has been observed in polycythemia vera (PV) and *JAK2*V617F mutation positive versus negative primary myelofibrosis (PMF) patients. Moreover, thrombocytosis was reduced by *JAK2*V617F allele burden in essential thrombocythemia (ET) and PMF. 261 significantly changed genes have been detected in PV, 82 in ET, and 94 genes in PMF. The following JAK-STAT signaling pathway related genes had augmented expression in CD34^+^ cells of MPNs: *CCND3* and *IL23A* regardless of *JAK2*V617F allele burden; *CSF3R, IL6ST*, and *STAT1/2* in ET and PV with *JAK2*V617F mutation; and *AKT2, IFNGR2, PIM1, PTPN11*, and *STAT3* only in PV. *STAT5A* gene expression was generally reduced in MPNs. IL-6 cytokine levels were increased in plasma, as well as IL-6 protein levels in bone marrow stroma of MPNs, dependent on *JAK2*V617F mutation presence in ET and PMF patients. Therefore, the *JAK2*V617F mutant allele burden participated in inflammation biomarkers induction and related signaling pathways activation in MPNs.

## 1. Introduction

It has been generally accepted that chronic inflammation may increase the risk of cancer by providing bioactive molecules from the tumor microenvironment, including cytokines and growth factors that promote continuous cell proliferation and malignant transformation [1]. Interleukin-6 (IL-6) is a member of the IL-6-type cytokines family with pro- and anti-inflammatory properties. They activate the Janus tyrosine kinase (JAK) family members (JAK1, JAK2, and TYK2), leading to the activation of transcription factors of the signal transducer and activator of transcription (STAT) family. All IL-6-type cytokines potently activate STAT3 and to a minor extent STAT1, while SOCS3 is the primary inhibitor of IL-6 signaling [2, 3]. IL-6 is produced by a wide range of cells including monocytes/macrophages, T cells, endothelial cells, fibroblasts, and hepatocytes. IL-6 regulates a variety of cell functions such as a cell proliferation, activation, and differentiation and has been proposed as a predictor of malignancy [4, 5].

IL-6 has been implicated as a critical activator of myelopoiesis in response to chronic inflammatory disorders, including myeloproliferative neoplasm (MPN) [6]. IL-6 levels are increased in primary myelofibrosis (PMF), with a positive correlation between IL-6 and angiogenesis in bone marrow of MPN patients [7, 8]. The endogenous levels of IL-6 and soluble IL-6 receptor are also elevated in essential thrombocythemia (ET) [9]. It has been reported that although serum level of IL-6 is not increased in polycythemia vera (PV), the increased percentage of megakaryocytes secreting this cytokine is observed in bone marrow of patients with PV [10].

Activation of the JAK-STAT signaling pathway resulted in the production of IL-6 [11]. Moreover, JAK2-specific inhibition blocks STAT3 phosphorylation and IL-6 production in cancer cells [12]. STAT3 is predominantly activated by IL-6 receptor via JAK1/2 in cancer cell lines in association with transcriptional silencing of SOCS-1 by hypermethylation [13]. Moreover, JAK1/2 inhibition decreases a circulating level of proinflammatory cytokine IL-6 in MPN mouse model [14]. In a transgenic mouse model of Jak2V617F-mediated MPN, JAK2 inhibitor normalizes the pathologically high plasma concentrations of IL-6 [15]. Tumor necrosis factor-alpha (TNF-*α*) stimulated IL-6 production is also suppressed by inhibition of JAK2 in multiple myeloma cells [16].

According to previous reports, proinflammatory cytokine IL-6 demonstrates JAK-STAT signaling dependence and has been augmented in MPNs. Majority of MPN patients have* JAK2*V617F mutation that constitutively stimulates JAK-STAT pathway. Using microarray analysis, we explore both JAK-STAT and IL-6 signaling related genes in MPNs according to* JAK2*V617F mutant allele burden. IL-6 levels have been examined both in peripheral blood and bone marrow of patients with MPN. In addition, genes related to other major inflammatory pathways, such as transforming growth factor-beta (TGF-*β*) and nuclear factor-kappa B (NF-*κ*B), have been evaluated in MPNs.

## 2. Material and Methods

### 2.1. Isolation of CD34^+^ Cells from the Peripheral Blood of MPN Patients

The study was approved by the Local Research Ethics Committee (Medical School, University of Belgrade). Informed consent was obtained from all patients included in the study. All study de novo patients were subject to 30 mL of peripheral blood draw on one occasion, collected in 10% sodium citrate. Each 30 mL of diluted peripheral blood (1 : 1.2 with Ca^2+^/Mg^2+^-free phosphate buffer saline (PBS)) was layered gently on top of 15 mL lymphocyte separation medium (Capricorn Scientific GmbH, Ebsdorfergrund, Germany). After centrifugation (400 g, 30 min, 20°C), the interface of containing mononuclear cells was collected and washed with PBS. The CD34^+^ cells were isolated from the collected mononuclear cells by magnetic separation column (Super Macs II, Miltenyi Biotec, Bergisch Gladbach, Germany) and mixture of magnetic microbeads conjugated with antibody against CD34 (Miltenyi Biotec) according to the manufacturer's instructions. The viable CD34^+^ cell counts were performed with the use of a trypan-blue exclusion technique (BioWhittaker). The high purity of recovered CD34^+^ cells was determined by flow cytometry using PE-anti-CD34 mAb (BD Biosciences, San Jose, CA, USA). Karyotype analysis did not show any chromosome aberrations in samples for microarray analysis.

### 2.2. Isolation of Total RNA

We used the RNeasy protocol for isolation of total RNA from CD34^+^ cells according to the manufacturer's instructions (Qiagen GmbH, Hilden, Germany). Concentration and integrity of total RNA were assessed using NanoDrop spectrophotometer (Thermo Fisher Scientific Inc., Wilmington, Delaware, USA) and Agilent 2100 Bioanalyzer Software (Agilent Technologies, Waldbronn, Germany) comparing the ratio of 28S and 18S RNA peaks to ensure that there is minimal degradation of the RNA sample.

### 2.3. DNA Sequencing

Genomic DNA was extracted from peripheral blood granulocytes of MPN patients by using the proteinase K and phenol-chloroform technique. Single nucleotide mutation of* JAK2*V617F gene was characterized by DNA sequencing after PCR amplification. PCR amplification was performed with wild-type JAK-2-specific forward primer 5′-tggcagagagaattttctgaact-3′ and reverse primer 5′-ttcattgctttcctttttcaca-3′, confirmed by electrophoresis on an ethidium bromide-impregnated 1% agarose gel. PCR amplified samples were analyzed by sequencing on an automated ABI PRISM 3130 Genetic Analyzer (Applied Biosystems; Life Technologies, Carlsbad, CA, USA) with AB DNA Sequencing Analysis Software (v5.2) by using the Big Dye Terminator v3.1 Ready Reaction Cycle Sequencing Kit.

### 2.4. Immunohistochemical Analysis

Bone marrow tissue sections were fixed in 10% neutral formalin solution for 24–36 hours and then decalcified in EDTA buffer for 3 hours and embedded in paraffin. The tissue sections were cut at 5 *μ*m, heated at 56°C for 60 min, and then deparaffinized and rehydrated through a series of xylenes and alcohols followed by an epitope retrieval step. Samples were treated with 3% H_2_O_2_ solution in PBS to block endogenous peroxidase activity. The next step was incubation with anti-IL-6 antibody (Thermo Scientific, dilution 1 : 400) in a humidity chamber overnight at room temperature. Immunostaining was performed using the streptavidin-biotin technique (LSAB+/HRP Kit, DAKO). Immunoreactivity was visualized with DAKO Liquid DAB+ Substrate/Chromogen System counterstained with Mayer's hematoxylin (Merck, Whitehouse Station, NJ). For the negative control samples, normal serum and PBS buffer (1 : 500) were pipetted without primary antibodies. Immunoreactive cells were analyzed and scored at five powered fields in each sample using a computer-supported imaging system (analysis Pro 3.1) connected to the light microscope (Olympus AX70, Hamburg, Germany) with an objective magnification of ×40. Immunohistochemical staining for IL-6 was performed in bone marrow of 34 subjects (10 PV: per 5 heterozygous and homozygous; 9 ET: 5 no mutation and 4 heterozygous, and 9 PMF: 5 no mutation, 4 heterozygous for* JAK2*V617F, and 6 healthy subjects).

### 2.5. ELISA Assay

Peripheral blood samples were obtained from 45 patients with newly diagnosed, untreated MPNs. The study included 14 PV, 14 ET, and 17 PMF cases. Patients were subclassified according to* JAK2*V617F mutational status as JAK2 homozygous, JAK2 heterozygous, and patients without mutation. We analyzed 7 PV homozygous and 7 PV heterozygous patients, 7 ET heterozygous and 7 ET patients without* JAK2*V617F mutation, and 3 PMF homozygous, 7 PMF heterozygous, and 7 PMF patients without* JAK2*V617F mutation. The control group comprised 7 healthy volunteers. Blood samples were collected with EDTA, and plasma was separated by centrifugation at 2000 rpm per 15 minutes. The samples were stored at −80°C until analysis. Plasma IL-6 level was measured using an ELISA kit (Thermo Scientific, Rockford, IL, USA), according to the manufacturer's instructions. All samples were tested in duplicate and data were expressed as average of IL-6 levels in pg/mL for each group. Measurements were performed on an ELISA Multiscan Plus plate reader (Labsystems, Finland). Data were expressed as mean ± standard error of the mean (SEM) of each group. They were analyzed by Student's* t*-test. A *p* value < 0.05 was chosen as the level of statistical significance.

### 2.6. Microarray Analysis

The human oligo probe set was purchased from Operon Human Genome Array-Ready Oligo Set Version 4.0 (Eurofins MWG Operon, Huntsville, AL, USA) which contains 35.035 oligonucleotide probes, representing approximately 25.100 unique genes. We have followed the MIAME (minimum information about a microarray experiment) guidelines for the data presentation. Also, our prior experience with primary cell cultures included quantitative PCR with housekeeping genes (S16 and HPRT) to establish similar efficiency of cDNA synthesis and PCR (data not shown). In microarray studies, for determination of gene expression in CD34^+^ cells of MPNs we used 8 healthy donors and 9 ET (4 negative and 5 positive heterozygotes for* JAK2*V617F mutation), 7 PV (3 heterozygotes and 4 homozygotes for* JAK2*V617F), and 4 PMF (2 negative and 2 positive for* JAK2*V617F) patients. We isolated a low quantity of CD34^+^ cells that correspond to low mRNA levels insufficient for microarray analysis, so we performed the amplification of total RNA using the Amino Allyl MessageAmp II aRNA Amplification kit (Life Technologies Corp., Carlsbad, CA, US), according to manufacturer instruction. We used 0.3 *μ*g of total RNA from MPN patients for amplification. For microarray analyses we used 3 *μ*g of amplified RNA. Total human universal RNA (HuURNA), isolated from a collection of adult human tissues to represent a broad range of expressed genes from both male and female donors (BD Biosciences, Palo Alto, CA), served as a universal reference control in the competitive hybridization. All examined MPN samples were hybridized against HuURNA. For hybridization, the hybridization mixture of cDNA probe and aRNA was preheated at 100°C for 2 minutes and centrifuged for 1 minute at 10.000 rpm. 20 *μ*L of preheated (42°C) Ambion hybridization buffer (20x SSC in 10% SDS) was mixed with hybridization mixture. Total volume of the hybridization mixture was added on the array in slide and covered with cover slip. Slides were placed in MAUI hybridization chamber (BioMicro Systems, Inc., Salt Lake City, UT, USA) and incubated overnight at 42°C. Slides were then washed for 4 minutes each in 1x SSC and 0.1x SSC and spin-dried. Microarray slides were scanned in both Cy3 (532 nm) and Cy5 (635 nm) channels using Axon GenePix 4000B scanner (Axon Instruments, Inc., Foster City, CA) with a 10-micron resolution. Scanned microarray images were exported as TIFF files to GenePix Pro 3.0 software for image analysis. The average of the total Cy3 and Cy5 signal gave a ratio that was used to normalize the signals. Each microarray experiment was globally normalized to make the median value of the log_2_-ratio equal to zero. For advanced data analysis, gpr and jpeg files were imported into microarray database and normalized by software tools provided by NIH Center for Information Technology (http://nciarray.nci.nih.gov/). Spots with confidence interval of 99 (≥2 fold) with at least 150-fluorescence intensity for both channel and 30 *μ*m spot size were considered good quality spots for analysis. The microarray data were available from Gene Expression Omnibus (http://www.ncbi.nlm.nih.gov/geo; accession number GSE55976).

### 2.7. Statistical Analysis

For microarray data management and analysis, we used NCI/CIT microarray database (mAdb) system. The one-way ANOVA was applied using mAdb software for measurement of statistical significance in gene expression among MPNs. For mAdb hierarchical clustering we used uncentered correlation that applied a modified Pearson correlation equation which assumes that the means are 0.

## 3. Results

### 3.1. Analysis of Peripheral Blood Cells in MPN Patients according to* JAK2*V617F Mutant Allele Burden

We examined the levels of peripheral blood cells from MPN patients in correlation to* JAK2*V617F mutant allele burden. We observed 63 patients with ET, 92 patients with PV, 50 patients with PMF, 12 healthy subjects, and 10 patients with secondary erythrocytosis. Level of thrombocytes was reduced, while erythrocyte and hemoglobin levels were elevated in ET patients heterozygous for* JAK2*V617F mutation versus ET patients without JAK mutation (Table 1). PV patients heterozygous and homozygous for* JAK2*V617F mutation had significantly increased levels of CD34^+^ cells, leukocytes, thrombocytes, and erythrocytes but reduced MCV compared to secondary erythrocytosis. CD34^+^ cells were generally increased in peripheral blood of PMF patients, particularly in* JAK2*V617F mutation positive patients. The average number of CD34^+^ cells (cells/*μ*L) in peripheral blood was approximately 6 in PV and ET and 111 in PMF group, increased in comparison to mean value for healthy subjects (2.75 ± 1 cells/*μ*L). In addition, leukocytes and erythrocytes were increased both in heterozygous and homozygous* JAK2*V617F mutated forms comparing to PMF patients without mutation. We observed lower thrombocyte levels and MCV in homozygous PMF patients, while hemoglobin levels were significantly increased in heterozygous PMF patients compared to* JAK2*V617F unmutated PMF patients (Table 1).

### 3.2. Microarray Analysis of Total Gene Expression in CD34^+^ Cells of MPNs according to* JAK2*V617F Mutant Allele Burden

**Figure 1:**
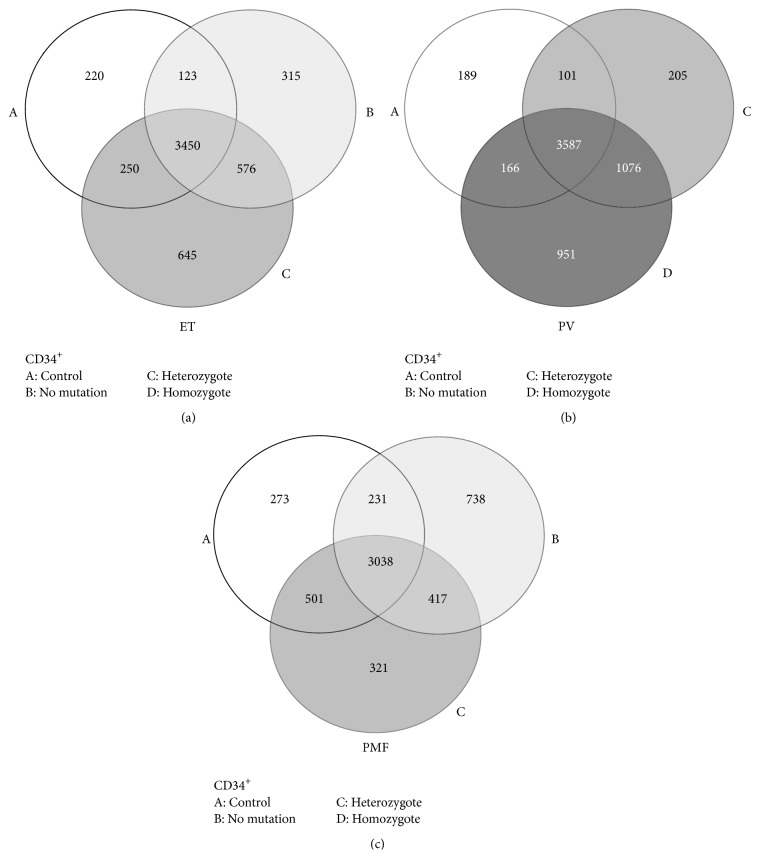
Microarray study of gene expression in CD34^+^ cells from peripheral blood of MPN patients. The Venn diagram shows similarity of total gene expression in controls (*n* = 8) and (a)* JAK2*V617F heterozygous and no mutation ET forms (*n* = 9), (b)* JAK2*V617F heterozygous and homozygous PV forms (*n* = 7), and (c)* JAK2*V617F heterozygous and no mutation PMF forms (*n* = 4).

Using microarray analysis, the total gene expression has been examined separately in CD34^+^ cells of PV, ET, and PMF groups. A distribution of total gene expression of MPN CD34^+^ cells has been presented in comparison to controls and according to* JAK2*V617F mutant allele burden (Figure 1). The quantity of shared genes in CD34^+^ cells was presented by Venn diagram in* JAK2*V617F heterozygous, homozygous, and mutation-free forms (Figure 1). Moreover, the statistical analyses of total gene expression have been performed in PV, ET, and PMF according to* JAK2*V617F mutant allele burden (see Supplemental Table 1 in Supplementary Material available online at http://dx.doi.org/10.1155/2015/453020). We observed 261 significantly changed genes (*p* < 0.01) in PV, 82 significant genes (*p* < 0.01) in ET, and 94 genes (*p* < 0.05) in PMF comparing to controls and sporadically among MPNs (Supplemental Table 1). ET and PMF shared 43% and 26% of their significantly changed genes with PV, respectively. CHMP5 gene expression was generally increased in MPNs and further on augmented by* JAK2*V617F allele burden. SOD2 gene expression was also generally increased in MPNs but only in PV augmented by* JAK2*V617F allele burden. WAC gene expression was generally increased in PV, ET, and heterozygous PMF patients. CCND3, HN1, and LOC124685 were only significantly upregulated genes simultaneously in ET and PMF compared to control subjects (Supplemental Table 1).

### 3.3. JAK-STAT Signaling Pathway Related Genes in CD34^+^ Cells of MPNs

**Figure 2:**
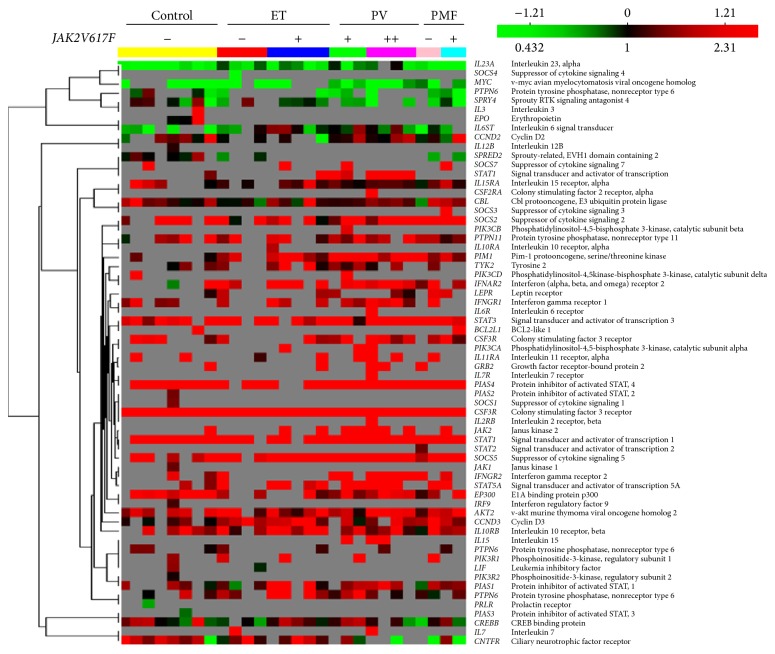
Hierarchical clustering of JAK-STAT signaling pathway related genes expressed in CD34^+^ cells of MPNs. The color indicates the relative fold expression of each gene: red indicates increased expression, green negative expression, and black not changed expression, while gray stands for absent expression per each examined sample. The gene correlations are uncentered.

The examination of* JAK2*V617F mutant allele burden influence has been expanded toward JAK-STAT signaling pathway in CD34^+^ cells of MPNs. According to Table 2, several genes involved in JAK-STAT signaling have been modified by* JAK2*V617F allele burden. Colony stimulating factor 3 receptor (*CSF3R*) has been significantly increased in* JAK2*V617F positive ET and PV patients. The v-akt murine thymoma viral oncogene homolog 2 (*AKT2*) has been significantly increased in* JAK2*V617F homozygous PV and* JAK2*V617F positive PMF patients. Cyclin D3 (*CCND3*) has been generally augmented in MPNs not influenced by* JAK2*V617F mutation. Pim-1 oncogene (*PIM1*) has been also generally augmented in MPNs but reached statistical significance only in PV, similar to protein tyrosine phosphatase, nonreceptor type 11 (*PTPN11*). However,* PTPN6* has been significantly increased only in* JAK2*V617F heterozygous ET patients.* STAT1* and* STAT2* gene expression have been increased in heterozygous ET and PV patients, while* STAT3* only in homozygous PV patients.* STAT5A* has been significantly decreased in ET and PMF with no* JAK2*V617F mutation as well as in homozygous PV patients (Table 2). In addition, these JAK-STAT signaling related genes were also shown in hierarchical clustering analysis to illustrate their associations (Figure 2). The genes only and steadily expressed in homozygous PV were* GRB2, IL15, LEPR*, and* PIK3CA* (Table 2).

### 3.4. IL-6 and Inflammatory Signaling Pathways Related Gene Expression in CD34^+^ Cells of MPNs

Regarding IL-6 signaling pathway related genes several of them were already presented in Table 2, such as* PTPN11, STAT3, JAK1, JAK2*, interleukin 6 signal transducer (*IL6ST*), and growth factor receptor-bound protein 2 (*GRB2*).* IL6ST* has been increased in CD34^+^ cells of* JAK2*V617F mutation positive ET and PV patients (Table 2).* MAP2K1* was significantly increased in CD34^+^ cells of PV (Table 3).* RAF1* and* FOS* gene expression were increased in ET and PV but do not reach statistical significance compared to controls (Table 3). Other proinflammatory pathways such as NF-*κ*B and TGF-*β* signaling demonstrated elevation of* TNFRSF1A* and* TRADD* genes expression in CD34^+^ cells of PMF and PV, respectively, as well as decrease of* CDKN2B* and increase of* TFDP1* genes in PMF (Table 3). Anti-inflammatory IL-10 signaling pathway related genes* BLVRA* and* HMOX1* demonstrated increased expression in CD34^+^ cells of PV and ET, respectively (Table 3).

### 3.5. IL-6 Protein Levels in MPNs according to* JAK2*V617F Mutant Allele Burden

**Figure 3:**
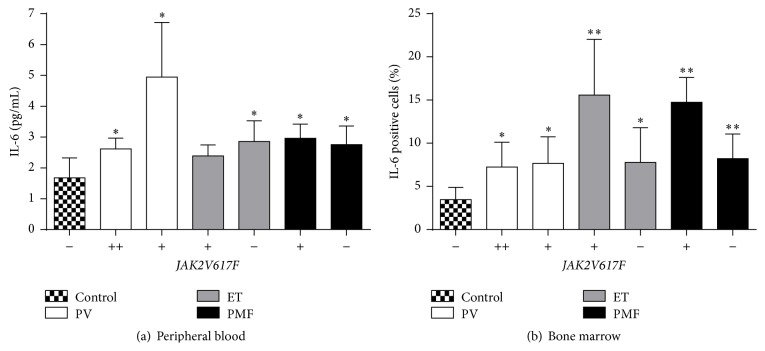
IL-6 levels in peripheral blood and bone marrow of MPNs in accordance with* JAK2*V617F mutant allele burden. (a) IL-6 level in peripheral blood of patients with MPN determined by ELISA (*n* = 7). (b) IL-6 level in bone marrow of patients with MPN determined by immunohistochemistry. (−) no* JAK2*V617F, (+) heterozygosity, and (++) homozygosity for* JAK2*V617F mutation (*n* = 4-5, plus control *n* = 6). Values are mean ± SEM. ^*∗*^ *p* < 0.05, ^*∗∗*^ *p* < 0.01 regarding MPNs versus Control.

We examined IL-6 protein levels in peripheral blood (plasma) and bone marrow of MPNs in accordance with* JAK2*V617F mutant allele burden. IL-6 protein levels were generally increased in plasma of MPNs reaching statistical significance in majority of patients, not influenced by* JAK2*V617F mutant allele burden (Figure 3(a)). Furthermore, there is a positive correlation between IL-6 protein levels and erythrocytes (*r* = 0.933, *p* < 0.01), hemoglobin (*r* = 0.733, *p* < 0.05), and hematocrit (*r* = 0.818, *p* < 0.05) levels in PV patients heterozygous for* JAK2*V617F mutation. IL-6 protein levels were also commonly and significantly increased in bone marrow stromal cells of MPNs (Figure 3(b)). This bone marrow augmentation of IL-6 expression was more prominent in* JAK2*V617F positive ET and PMF patients (*p* < 0.01, Figure 3(b)).

## 4. Discussion

Presence of* JAK2*V617F mutation correlated with a reduced level of thrombocytosis in ET and increased absolute number of circulating CD34^+^ cells, leukocytosis, and erythrocytosis in patients with PV. In addition, increased* JAK2*V617F allele burden augmented leukocytosis and erythrocytosis but reduced thrombocytosis and MCV in patients with PMF. Using Venn diagram, a distribution of gene expression has been presented, with a list of significantly modified genes in CD34^+^ cells of MPNs, compared to controls and according to* JAK2*V617F mutant allele burden. 261 significantly changed genes in PV have been observed, 82 in ET, and 94 genes in PMF. We demonstrated that majority of JAK-STAT signaling pathway related genes have been augmented in MPNs with increased* JAK2*V617F allele burden, except STAT5 gene. Proinflammatory IL-6 (*IL6ST, PTPN11*), NF-*κ*B (*TNFRSF1A, TRADD*), and TGF-*β* (*TFDP1*) signaling pathways demonstrated increase of the related genes, as well as certain anti-inflammatory IL-10 signaling pathway related genes (*BLVRA, HMOX1*) in CD34^+^ cells of MPNs. IL-6 cytokine levels were increased in plasma of MPNs, as well as IL-6 protein levels in bone marrow stroma of MPNs, where they demonstrated dependence of* JAK2*V617F presence in ET and PMF patients.

According to a large study with 1545 PV patients, median hemoglobin level was 184 g/L (in range 151–265), median leukocyte count was 10.4 × 10^9^/L (in range 3–171), and median platelet count was 466 × 10^9^/L (in range 7–2370). Leukocytosis was present in 49%, while thrombocytosis in 53% of PV patients [20]. In other study with 327 PV patients, median hemoglobin level was 176 g/L, median leukocyte count was 13 × 10^9^/L, and median platelet count was 515 × 10^9^/L [21]. In study with 707 PMF patients, median hemoglobin level was 110 g/L (in range 63–149), median leukocyte count was 13 × 10^9^/L (in range 2.7–35.0), and median platelet count was 515 × 10^9^/L (in range 44–990) [22]. In next study with 612 MPN patients, mean hemoglobin level was 137, 174, and 119 g/L, mean leukocyte count was 10.2, 12.4, and 13.3 × 10^9^/L, and mean platelet count was 889, 579, and 517 × 10^9^/L for ET, PV, and PMF patients, respectively [23]. According to a small study with 21 ET patients, median red cell level was 4.46 × 10^12^/L (3.14–5.78), median hemoglobin level was 135 g/L (105–166), median leukocyte count was 10.7 × 10^9^/L (5.1–19.8), and median platelet count was 872 × 10^9^/L (310–1443). In the same report with 17 PV patients, median red cell level was 6.15 × 10^12^/L (5.09–7.95), median hemoglobin level was 165 g/L (138–205), median leukocyte count was 10.0 × 10^9^/L (6.6–27.2), and median platelet count was 501 × 10^9^/L (221–936) [24]. Reported laboratory findings are in range of our presented results. In our study, hemoglobin mean level 158 g/L for PV was reduced, 142 g/L for ET was similar, 138 g/L for PMF was increased in comparison to other studies but is still in range of the observed results [20, 21, 24]. The levels of red blood cells and leukocytes in our MPN patients were similar to previous reports. The level of platelets in our ET patients was similar but elevated in PV and PMF patients in comparison to previous results. However, the increased values were within the limit of the reported results.

Median numbers and ranges of circulating CD34^+^ cells were 2.3 × 10^6^/L (0–5) in control subjects, 2.2 × 10^6^/L (0–14) in those with PV, 2.4 × 10^6^/L (0–14) in those with ET, and 114 × 10^6^/L (6–2520) in PMF patients [25]. Comparing to our study, CD34^+^ cell levels were reduced in ET and PV patients but were still within range, while they were similar for PMF patients (Table 1). More similar median levels of circulating CD34^+^ cells in PV and ET have been observed in another study: 8.9 × 10^6^/L (0–31) in those with PV, 4.1 × 10^6^/L (0–18) in those with ET, and 60 × 10^6^/L (11–449) in PMF patients [17]. A significant and positive correlation between the proportion of* JAK2*V716F mutant alleles and circulating CD34^+^ counts in MPNs has been found. CD34^+^ cell counts were similar in healthy subjects (median value 1.3 × 10^6^/L) and individuals with secondary erythrocytosis (median value 1.8 × 10^6^/L). However, PV patients with* JAK2*V617F had higher circulating CD34^+^ counts than healthy individuals without the mutation [26]. This is in accordance with our observation. PV patients carrying up to 50%* JAK2* mutant alleles had slightly but significantly higher CD34^+^ cell counts (median value 3.2 × 10^6^/L) than controls, doubled in our results. Moreover, patients with PV and more than 50%* JAK2*V617F allele had markedly higher CD34^+^ cell counts (median value 21.4 × 10^6^/L) than those with 50% or less mutant alleles [26]. This value for CD34^+^ cell level in PV was doubled in comparison to our result (9.4 × 10^6^/L) for PV patients with more than 50%* JAK2*V617F allele. In addition, according to our results ET patients regardless of* JAK2* mutant alleles had higher circulating CD34^+^ counts than healthy individuals. Moreover, PMF patients had also increased level of circulating CD34^+^ cells than healthy individuals, more elevated in patients with* JAK2*V617F allele.

Chronic inflammation may be an initiator of clonal evolution in patients with MPNs. The presence of elevated leukocyte and thrombocyte counts in MPNs may be the result of both clonal myeloproliferation and chronic inflammation presented by hypersensitivity to cytokine stimulation [27]. We demonstrated increased expression of JAK-STAT related genes and increased IL-6 levels in MPNs. IL-6-JAK1-STAT3 signal transduction pathway had an important role in the conversion of differentiated cancer cells into cancer stem cells [28]. Inhibition of JAK blocked IL-6-induced phosphorylation of STAT3 but failed to block the phosphorylation of mitogen-activated protein kinase (MAPK) [29]. Another major signaling pathway for IL-6-type cytokines was the MAPK cascade [2]. Soluble calreticulin induced proinflammatory cytokine IL-6 in macrophages via MAPK-NF-*κ*B signaling pathway [30]. SOCS1 expression was elevated in MPN granulocytes but the level was independent of* JAK2*V617F mutational status. SOCS3 is an inhibitor of IL-6 and STAT3, regulated by DNA methylation. The transcript levels of* SOCS3* were increased in granulocytes from* JAK2*V617F-positive MPN patients. Hypermethylation of the* SOCS3* promoter was identified in 32% of patients with PMF but not in patients with ET and PV [31].

We presented expression of genes related to inflammatory NF-*κ*B and TGF-*β* signaling in MPNs. IL-6 transsignaling was dependent on STAT3 and mediated through enhanced TGF-*β* signaling [4]. A dysregulation of NF-*κ*B was already addressed as responsible for the MPNs [31]. NF-*κ*B dysregulation in microRNA-146a-deficient mice drove the development of myeloid malignancies. MicroRNA-146a has been implicated as a negative feedback regulator of NF-*κ*B activation. The mice also exhibited chronic myeloproliferation in their bone marrow, with increased transcription of NF-*κ*B-regulated genes [32]. NF-*κ*B was a central mediator of TGF-*β* induction in monocytes from patients with PMF [33]. A spontaneous activation of NF-*κ*B was also detected in proliferating megakaryocytes and circulating CD34^+^ cells of patients with PMF, involved in TGF-*β*1 secretion [34]. Patients with MPN had higher peripheral blood plasma levels of both bioactive and total TGF-*β*1 compared to healthy controls [35]. TGFBR2 was significantly overexpressed in advanced PMF stages produced by endothelial cells of the increased microvessel network [36]. Expression of members of bone morphogenetic protein (BMP) family, BMP1, BMP6, BMP7, and BMP-receptor 2, was significantly increased in advanced stages of PMF [36]. Cytokine IL-10 had increased levels in plasma of PMF patients [8]. In our analysis, gene expression of receptor for IL-10 (*IL10RB*) was increased in CD34^+^ cells of ET and PV and reduced in PMF with no statistical significance.

The recent JAK1/2 inhibitor trials in MPNs unexpectedly showed that reducing inflammation can be more beneficial to patients than targeting gene mutants [37]. Increased level of cytokine IL-6 was associated with shorter survival in patients with PMF [8]. The proportion of hematopoietic cells expressing the* JAK2*V617F mutation decreased after in vitro differentiation of CD34^+^ cells in the presence of optimal concentration of IL-6 and other growth factors [38]. Panobinostat, a pan-deacetylase inhibitor that depletes* JAK2*V617F levels and JAK/STAT signaling in MPN cells, reduced IL-6 level in plasma of PMF patients [39]. Moreover, IL-6 secreted by stromal cells protected* JAK2*V617F mutated cells, MPN clones, from JAK2 inhibitor therapy [40]. Siltuximab, a chimeric anti-IL-6 monoclonal antibody, did not reduce RBC transfusions in transfusion-dependent patients with low- and intermediate-1-risk myelodysplastic syndrome [41]. The addition of siltuximab to bortezomib did not improve progression-free or overall survival regardless of a numerical increase in response rate of patients with relapsed or refractory multiple myeloma [42]. The addition of siltuximab to bortezomib-melphalan-prednisone improved the complete response rate by 5%, overall response rate by 8%, and partial response rate by 20% in multiple myeloma [43]. Siltuximab in combination with dexamethasone yielded a partial response rate of 23% in patients with relapsed or refractory multiple myeloma [44].

For microarray analyses of CD34^+^ cells we used amplified RNA. Polacek et al. observed 94% overlap of the differentially expressed genes when starting with 100 ng total RNA input in the amplification procedure [45]. Greater than 90% overlap has been reported with oligo arrays when starting with 250 ng total RNA input [46, 47]. In addition, the quality of the array data was superior to that obtained using total RNA [48]. Therefore, we used 300 ng total RNA input for amplification and it is couple times higher than or similar to the previous observations with more than 90% overlap. By this approach, we greatly reduced the possibility of biases in the relative representations of unique RNAs. Previous profile reports included various quantities of examined genes from purified CD34^+^ cells of PMF and PV subjects (Table 4) [17–19]. We performed microarray studies with 25.100 genes in CD34^+^ cells from the peripheral blood of MPN subjects. According to different technical approaches, as presented in Table 4 (we prepared amplified RNA from peripheral blood of individual MPN patients and controls rather than performing pools of RNA from patient groups and bone marrow derived CD34^+^ cells), we found some minor overlap in differentially expressed genes. In microarray study of well characterized and significantly different genes performed by Guglielmelli et al.,* FGFR1*,* KLF4*, and* TGFBI* levels were decreased, while* IFITM1* level was increased in PMF CD34^+^ cells [17]. In our study,* FGFR4*,* KLF2* gene expression was increased in PV, augmented by* JAK2*V617F mutant allele burden, while* TGFBI* level was increased in PV and ET patients.* IFITM1* level was also increased in our PV and ET CD34^+^ cells, enhanced by* JAK2*V617F mutant allele burden (Supplemental Table 1). In microarray study of Jones et al.,* TNFRSF1A* and* TGIF2-C20ORF24* were downregulated, while* NCF2* gene expression was upregulated in PMF CD34^+^ cells [18]. In our study,* NCF2* gene expression was upregulated in PV, while* TNFRSF1A* was upregulated in* JAK2*V617F positive ET patients. In gene expression profiling study by Steidl et al.,* CAPNS1*,* CASP1*,* GPX1*, and* HSPA8* were downregulated, while* FGFR4*,* TGFBI*, and* IL-6* were upregulated in PV CD34^+^ cells [19]. In our study,* CAPNS1* and* HSPA8* were downregulated in PMF and PV, whereas* CASP1*,* IL6ST*, and* GPX1* were upregulated in PV and ET, respectively;* FGFR4* was upregulated in PV patients, augmented by* JAK2*V617F allele burden. Therefore, the increased levels of inflammation biomarkers (*TGFBI, IL-6*) were also detected in previous microarray studies.

## 5. Conclusion

Besides determination of significantly changed genes in CD34^+^ cells of MPNs, we confirmed activation of JAK-STAT signaling related genes, such as* CSF3R, IL6ST, PIM1*, and* STAT1/2*. Regarding parameters of inflammation, leukocytosis was increased in PV and PMF, while thrombocytosis was reduced in ET and PMF with* JAK2*V617F mutation. Proinflammatory cytokine IL-6 levels were generally increased in peripheral blood and bone marrow of patients with MPN but were more prominent in* JAK2*V617F mutation positive ET and PMF patients. Therefore,* JAK2*V617F mutant allele burden participated in inflammation biomarkers induction. The presented inflammation biomarkers will provide a better understanding of molecular mechanisms implicated in the development and progression of MPNs.

## Supplementary Material

The statistical analyses of total gene expression in MPNs according to *JAK2V617F* mutant allele burden. We observed 261 significantly changed genes in PV, 82 significantly changed genes in ET, and 94 genes in PMF comparing to healthy subjects.

## Figures and Tables

**Table 1:** Blood test results of examined MPN patients according to *JAK2* mutant allele burden.

MPN	*JAK2*V617P	CD34^+^ (cells/*μ*L)	Leukocytes (×10^9^/L)	Thrombocytes (×10^9^/L)	Erythrocytes (×10^12^/L)	MCV	Hemoglobin (g/L)
ЕТ	0	6.3 (±3.4)	8.77 (±2.4)	1110 (±358)	4.6 (±0.4)	88.7 (±3.7)	134.1 (±14)
	<50%	6.2 (±6.1)	9.1 (±3.5)	815 (±249)^2^	5 (±0.53)^2^	87 (±3.9)	142 (±13)^1^

PV	Sec Er	2.5 (±0.7)	7.25 (±1.9)	203 (±46)	5.34 (±0.4)	89.8 (±3.6)	161.6 (±13)
	<50%	6.3 (±4.6)^1^	12.2 (±3.9)^2^	807 (±349)^2^	6.1 (±0.8)^2^	82 (±8.8)^2^	158.4 (±15)
	>50%	9.4 (±8.2)^1^	17.3 (±9.5)^2^	631 (±270)^2^	6.5 (±0.9)^2^	74.6 (±8.8)^2^	152.4 (±17)

PMF	0	29.8 (±37)	8.8 (±3.1)	905 (±320)	4.4 (±0.44)	87.1 (±5.6)	126.5 (±17)
	<50%	99 (±212)	12.9 (±5.1)^2^	804 (±364)	4.9 (±0.8)^1^	84 (±6.2)	138 (±14)^1^
	>50%	126 (±158)	14.2 (±5.5)^2^	509 (±331)^2^	5.34 (±1.3)^2^	77.7 (±11)^2^	129.1 (±20)

*t*-test: ^1^ *p* < 0.05, ^2^ *p* < 0.01 versus MPNs without JAK2 mutation (0) and secondary erythrocytosis (Sec Er).

**Table 2:** Statistically significant genes related to JAK-STAT pathway in CD34^+^ cells of MPNs according to *JAK2*V617F mutant allele burden.

	Control	ET		PV		PMF	
JAK2	−	−	+	Hetero	Homo	−	+
Genes	AV ± SD	AV ± SD	AV ± SD	AV ± SD	AV ± SD	AV ± SD	AV ± SD
**AKT2 **	0.94 ± 0.18	1.11 ± 0.58	0.80 ± 0.40	0.66 ± 0.37	1.3 ± 0.1^*∗∗*^	0.7 ± 0.30	1.1 ± 0.17^*∗*^
**CSF3R **	2.83 ± 0.20	2.99 ± 0.37	3.8 ± 0.6^*∗∗*^	3.2 ± 0.6^*∗*^	3.2 ± 0.2^*∗∗*^	2.92 ± 1.01	3.20 ± 0.50
**CBL **	0.49 ± 0.45	−0.1 ± 0.3^*∗*^	0.28 ± 0.65	0.29 ± 0.06	0.61 ± 0.14	0.16 ± 0.85	0.79 ± 0.13
CCND2	0.30 ± 0.55	0.01 ± 0.05	−0.38 ± 0.6	0.56 ± 0.39	0.38 ± 0.38	0.02 ± 0.20	0.33 ± 0.86
**CCND3 **	0.21 ± 0.22	1.1 ± 0.5^*∗∗*^	0.9 ± 0.4^*∗∗*^	0.66 ± 0.57	0.9 ± 0.3^*∗∗*^	1.2 ± 0.4^*∗∗*^	0.76 ± 0.2^*∗*^
CNTFR	0.52 ± 0.99	0.65 ± 0.83	0.97 ± 0.71	0.25 ± 0.47	−1.03 ± 0.2	−1.2	−0.64 ± 1.3
CREBBP	0.23 ± 0.60	−0.35 ± 0.3	0.35 ± 0.34	0.45 ± 0.44	0.10 ± 0.51	−0.41 ± 0.6	0.05 ± 0.58
GRB2	1.65		2.05		1.91 ± 0.12	1.48	
IFNAR2	0.51 ± 1.13	1.41 ± 0.46	1.76 ± 0.49	1.38 ± 0.25	1.32 ± 0.38	1.23 ± 0.69	0.83
IFNGR1	0.77 ± 0.22	0.92	1.14 ± 0.28	0.85 ± 0.12	1.18 ± 0.40	1.88	
**IFNGR2 **	0.77 ± 0.13	2.06	1.58 ± 0.60	1.94 ± 0.48	2.0 ± 0.1^*∗∗*^	1.96	1.87
IL10RB	0.96 ± 0.28	0.88 ± 0.55	1.52 ± 0.60	1.23 ± 0.49	0.99 ± 0.79	0.86 ± 1.01	0.80 ± 0.05
IL11RA	1.69 ± 0.13	0.31	1.87	1.66 ± 0.15	1.92 ± 0.39	1.49	
IL15			1.1	2.14	2.24 ± 0.04		
IL15RA	0.76 ± 0.47	0.19 ± 0.09	0.50 ± 0.34	0.27 ± 0.19	0.41 ± 0.07	0.16 ± 0.32	0.60 ± 0.39
**IL23A **	−1.18 ± 0.2	−0.7 ± 0.4^*∗*^	−1.11 ± 0.4	−0.75 ± 0.4	−0.5 ± 0.4^*∗*^	−1.3 ± 0.2^*∗*^	−1.7 ± 0.02^*∗∗*^
**IL6ST **	−1.10 ± 0.2	−0.46 ± 0.4	0.1 ± 0.6^*∗∗*^	0.06 ± 0.4^*∗*^	−0.1 ± 0.6^*∗*^	−0.92 ± 0.7	−0.84
**JAK1 **	0.88 ± 0.24	1.2	0.93	1.42 ± 0.03	1.7 ± 0.1^*∗∗*^	1.47	1.11
JAK2	2.25		3.54 ± 0.81	4.07 ± 0.27	3.96 ± 0.57		3.01
LEPR	0.18	0.92	0.77	0.81	0.39 ± 0.21	1.13	1.07
MYC	−1.86 ± 0.3	−2.28 ± 0.2	−1.70 ± 0.4	−1.27 ± 0.7	−1.58 ± 0.5	−1.70 ± 1.1	−0.79
PIAS1	0.48 ± 0.38	0.04 ± 0.49	1.10 ± 0.61	1.04 ± 0.37	0.97 ± 0.12	0.21 ± 0.69	0.61 ± 0.05
PIAS4	1.88 ± 0.43	2.05 ± 0.20	2.24 ± 0.77	1.86 ± 0.42	1.84 ± 0.12	1.89 ± 0.12	2.06 ± 0.51
PIK3CA			1.45	1.51	1.64 ± 0.13		
PIK3R1	0.72	1.04	2.41	2.45 ± 0.68	1.95	2.21	
**PIM1 **	0.28 ± 0.2	0.90 ± 0.19	1.10 ± 0.48	1.37 ± 0.4^*∗*^	1.42 ± 0.4^*∗*^	1.03 ± 0.38	0.82 ± 0.28
**PTPN11 **	0.78 ± 0.53	1.10 ± 0.46	0.84 ± 0.38	1.09 ± 0.43	1.03 ± 0.4^*∗*^	0.91	0.44 ± 0.16
**PTPN6 **	0.44 ± 0.43	0.70 ± 0.18	1.35 ± 0.4^*∗*^	1.12 ± 0.60	0.41 ± 0.19	0.05 ± 0.23	0.47
SOCS2	1.48 ± 0.61	1.12 ± 0.94	1.21 ± 0.87	1.71 ± 0.73	1.75 ± 1.09	2.06 ± 0.04	2.20 ± 0.90
SPRED2	0.17 ± 0.34	−0.45 ± 0.2	−0.58			−0.82	−0.73
SPRY4	−0.13 ± 0.7	−0.14 ± 0.7	−0.35 ± 0.2	−0.61 ± 0.2	−1.14 ± 0.2	−1.03	−1.00 ± 0.7
**STAT1 **	3.60 ± 0.55	3.37 ± 0.80	4.68 ± 0.8^*∗*^	4.4 ± 0.05^*∗*^	5.1 ± 0.5^*∗∗*^	3.31 ± 1.44	4.18 ± 0.39
**STAT2 **	1.45 ± 0.51	1.57 ± 0.38	2.39 ± 0.6^*∗*^	2.25 ± 0.2^*∗*^	2.01 ± 0.42	1.58 ± 0.74	1.75 ± 0.35
**STAT3 **	1.13 ± 0.26	1.54 ± 0.07	1.32 ± 0.30	1.05 ± 0.81	1.8 ± 0.3^*∗∗*^	1.59 ± 0.13	1.62 ± 0.57
**STAT5A**	1.39 ± 0.25	0.8 ± 0.01^*∗*^	0.90 ± 0.53	0.99 ± 0.43	1.01 ± 0.2^*∗*^	0.5 ± 0.2^*∗∗*^	1.93
TYK2	0.36 ± 0.28	0.63	0.85 ± 0.43	0.75 ± 0.33	0.35 ± 0.32	0.58	

^*∗*^ *p* < 0.05, ^*∗∗*^ *p* < 0.01, and gene expression in MPNs versus control.

**Table 3:** Inflammation signaling related gene expression in CD34^+^ cells of MPNs.

	Genes	Control		ET		PV		PMF	
		Mean	SD	Mean	SD	Mean	SD	Mean	SD
IL-6 signaling pathway related gene expression									
CEBPB	CCAAT/enhancer binding protein *β*	−0.3	0.48	−0.5	0.63	−0.3	0.49	−0.1	0.54
FOS	FBJ murine osteosarcoma viral oncogene homolog	2.46	0.87	3.11	1.04	3.74	0.95	2.35	1.38
MAP2K1	Mitogen-activated protein kinase kinase 1	0.49	0.31	0.7	0.15	**1.06**	**0.26**	0.19	0.52
RAF1	v-raf-1 murine leukemia viral oncogene homolog 1	1.66	0.29	2.02	0.27	2.11	0.34	1.95	0.38
SHC1	Src homology 2 domain cont transforming protein 1	−0.7	0	−0.3	0.54	0.27	0.04	−1.1	0
CSNK2A1	Casein kinase 2, *α* 1 polypeptide	0.23	0.06	0.23	0.05	0.35	0.51	0.48	0.52

NF-*κ*B signaling pathway related gene expression									
TNFRSF1A	**Tumor necrosis factor receptor superfamily, member 1A **	0.58	0.72	1.26	0.86	1.54	0.42	**1.64**	**0.40**
NFKBIA	Nuclear factor of kappa light polypept gene enhancer in B-cells inhibitor, *α*	4.24	0.82	3.70	1.02	3.57	0.91	2.82	1.06
TRADD	**TNFRSF1A-associat via death domain **	1.30	0.42	1.67	0.22	**1.79**	**0.31**	1.38	0.32
NFKB1	Nuclear factor of kappa light polypept gene enhancer in B-cells 1	0.69	0.17	0.28	1.13	1.17	0.24	1.34	1.16

TGF-beta signaling pathway related gene expression									
CDKN2B	**Cyclin-dependent kinase inhibitor 2B**	0.64	1.05	−0.1	1.15	−0.7	1.11	**−1.2**	**1.14**
ID3	**Inhibitor of DNA binding 3, dominant negative helix-loop-helix protein**	−2.1	0.48	−2.4	0.65	**−3.1**	**0.14**	−3.1	0.37
TFDP1	**Transcription factor Dp-1 **	−0.1	0.49	0.11	0.4	0.61	0.4	**0.96**	**0.69**
BMPR2	Bone morphogenetic prot rec type II	1.99	0	1.02	0.89	2.01	0.16		
TGFBR1	Transforming growth factor, *β* recep 1			0.59	0.35	1.04	0.5	0.66	0.14
TGFBR2	Transforming growth factor, *β* recep II	1.67	0.18			1.55	0.29	1.61	0

IL-10 signaling pathway related gene expression									
BLVRA	**Biliverdin reductase A**	0.70	0.59	1.13	0.45	**1.50**	**0.40**	1.31	0.29
IL10RB	Interleukin 10 receptor	0.96	0.31	1.30	0.70	1.15	0.73	0.83	0.75
HMOX1	**Heme oxygenase (decycling) 1**	1.30	0.44	**2.41**	**0.44**	2.32	0.59		

Bolded genes represent significantly changed expression versus controls (*p* < 0.05).

**Table 4:** Comparison of gene expression profiling between former and present studies in CD34^+^ cells of MPN subjects.

Studies	Guglielmelli et al. [17]	Jones et al. [18]	Steidl et al. [19]	Čokić et al.
MPN	PMF	PMF	PV	PV, ET, PMF

Purified from	PB-PMF BM-HC	PB-PMF BM-HC	BM-PMF BM-HC	PB-PV, ET, and PMF PB-HC

RNA	Pooled	Amplified 50 ng of total RNA	Individual	Amplified 300 ng of total RNA

Examined MPN patients	Mixed: de novo, treated/paused	Mixed: de novo, treated/paused	De novo	De novo

Number of patients	PMF, 3 × 5 HC, 3 × 5	PMF, 8 HC, 6	PV, 4 HC, 10	PV, 7, ET, 9, PMF, 4, and HC, 8

Microarray chips	Affymetrix HG-U133A GeneChip	Affymetrix HGU95Av2 chip	Atlas Clontech Human 1.2 I	Operon Human Genome 4.0

Number of examined genes	16.000 genes (22.238 probes)	9.670 genes (12.625 probes)	1.185 genes	25.100 genes (35.035 probes)

*JAK2*V617F	Mixture of 8 mutated and 7 nonmutated	Not analyzed	Not analyzed	Separated according to mutant allele burden

Characterized genes	174	95 (48 upregulated and 47 downregulated)	107	PV, 261, ET, 82, PMF, 94

HC: healthy controls, PB: peripheral blood, and BM: bone marrow.
