# Supplementary material for: Proinflammatory Cytokine IL-6 and JAK-STAT Signaling Pathway in Myeloproliferative Neoplasms
Source: Mediators Inflamm. 2015 Sep 29;2015:453020. doi: 10.1155/2015/453020 (PMC4602333; doi:10.1155/2015/453020)
Supplement: Supplementary file 1 — The statistical analyses of total gene expression in MPNs according to JAK2V617F mutant allele burden. We observed 261 significantly changed genes in PV, 82 significantly changed genes in ET, and 94 genes in PMF comparing to healthy subjects. [file 453020.f1.pdf]

Supplemental Table 1. The statistically significant genes among MPNs in hematopoietic $CD34^+$ progenitors determined by microarray analysis and related to *JAK2*V617F mutation. BG – Between Groups

PV		A		B		C		p value	Max. Mean Differ
*JAK2*V617F		Control		heterozygote		homozygote			
Gene	BG	Mean	SD	Mean	SD	Mean	SD		
LAMP1	B-A	-0.27	0.38	1.70	0.20	1.22	0.29	0.00001	1.97
ATP6V1B2	C-A	0.54	0.20	2.05	0.22	2.19	0.22	0.00001	1.65
CREBL2	C-A	0.26	0.26	1.07	0.23	1.37	0.20	0.00002	1.10
PARP9	C-A	0.62	0.21	2.35	0.45	2.64	0.24	0.00002	2.02
KIAA0930	B-A	1.37	0.46	3.13	0.32	2.56	0.47	0.00003	1.76
SOD2	C-A	1.74	0.17	2.45	0.26	2.69	0.40	0.00008	0.95
TYMP	C-A	2.24	0.40	3.74	0.00	4.23	0.05	0.00010	1.99
RNF213	C-A	0.45	0.04	0.99	0.08	1.72	0.10	0.00010	1.27
CHMP5	C-A	0.44	0.68	1.87	0.21	2.27	0.45	0.00011	1.83
SSU72	B-A	-1.06	0.23	-0.38	0.19	-0.53	0.14	0.00019	0.69
NPHP3	B-C	0.38	0.00	1.49	0.06	0.74	0.05	0.00019	0.75
DPF3	A-C	-0.84	0.10	-1.65	0.00	-2.12	0.19	0.00019	1.28
CORO1C	B-A	0.35	0.52	1.87	0.33	1.26	0.21	0.00019	1.53
FLJ11292	A-C	1.88	0.65	0.56	0.20	-0.66	0.30	0.00019	2.54
EGFL8	A-C	1.27	0.18	0.53	0.24	0.51	0.24	0.00021	0.76
LGALS9	A-B	1.10	0.18	-0.12	0.08	0.60	0.26	0.00022	1.22
MFSD1	C-A	1.52	0.49	2.66	0.31	2.75	0.30	0.00031	1.22
AFG3L1P	A-B	0.96	0.11	-0.33	0.10			0.00032	1.29
ATP1B3	C-A	-1.05	0.27	-0.68	0.00	-0.18	0.13	0.00034	0.86
PSMB9	C-A	1.49	0.44	2.64	0.28	2.84	0.45	0.00038	1.35
TGFBI	B-A	-0.31	0.34	1.24	0.36	0.27	0.58	0.00042	1.55
PTTG1IP	C-A	-0.75	0.24	-0.29	0.01	0.04	0.10	0.00043	0.79
LAP3	C-A	0.34	0.31	1.69	0.17	2.25	0.33	0.00048	1.92
SSX5	A-C	2.95	0.31	2.41	0.48	1.71	0.28	0.00050	1.24
FAM26F	C-A	2.73	0.69	4.11	0.36	4.77	0.51	0.00050	2.05
EIF4EBP2	B-A	1.17	0.32	2.57	0.65	1.85	0.22	0.00052	1.40
CDKN2D	C-A	1.80	0.44	2.81	0.22	3.17	0.42	0.00055	1.37
PDLIM5	B-A	1.02	0.41	2.21	0.26	2.04	0.23	0.00061	1.18
FAM127A	A-C	-0.28	0.55	-1.32	0.04	-1.44	0.09	0.00061	1.16
FLJ31306	C-A	1.37	0.18	1.61	0.19	2.18	0.10	0.00062	0.81
COPB2	B-A	0.22	0.48	1.30	0.41	1.21	0.18	0.00066	1.08
ST13P4	A-C	-0.77	0.28	-1.58	0.32	-1.63	0.13	0.00066	0.86
PRICKLE2-AS1	A-C	1.86	0.24	0.80	0.20	0.35	0.04	0.00071	1.50
STAT1	C-A	3.60	0.55	4.40	0.05	5.04	0.51	0.00073	1.45
CNTROB	A-C	3.08	0.32	2.32	0.57	2.08	0.11	0.00075	1.00
RAB8B	B-A	1.93	0.38	3.14	0.47	3.01	0.24	0.00077	1.21
KRTAP21-2	A-B	-0.91	0.19	-1.97	0.10	-2.47	0.00	0.00078	1.06
FLJ11292	A-C	1.76	0.59	0.90	0.35	-0.50	0.26	0.00080	2.26
GET4	A-B	-0.11	0.23	-0.88	0.11	-0.85	0.22	0.00082	0.77
UBE2D1	C-A	2.31	0.38	3.20	0.36	3.78	0.59	0.00084	1.47

GLRX	C-A	0.94	0.79	2.21	0.44	2.74	0.32	0.00086	1.80
WIBG	C-A	0.39	0.26	0.89	0.28	1.18	0.12	0.00088	0.79
TMEM60	C-A	-0.02	0.18	0.67	0.13	0.94	0.32	0.00090	0.96
NMI	C-A	1.91	0.69	3.20	0.33	3.34	0.30	0.00093	1.43
CEP89	A-C	-0.02	0.38	-0.98	0.25	-1.77	0.47	0.00093	1.75
SLTM	B-A	0.55	0.17	1.41	0.04	1.03	0.06	0.00096	0.85
NR2F6	A-C	1.05	0.34	0.63	0.22	0.18	0.18	0.00100	0.87
HSPA8	C-A	-1.03	0.60	-0.39	0.36	0.52	0.51	0.00100	1.55
ETNK1	C-A	0.39	0.23	1.25	0.36	1.42	0.15	0.00102	1.03
SLC27A3	C-A	1.35	0.54	2.39	0.27	2.51	0.40	0.00107	1.16
WAC	B-A	-0.20	0.41	0.88	0.36	0.57	0.27	0.00108	1.08
C1orf63	C-A	1.69	0.54	2.46	0.18	2.85	0.28	0.00109	1.16
DDB2	C-A	-0.44	0.09	-0.22	0.14	0.39	0.06	0.00112	0.82
MRPS18C	C-A	-0.44	0.43	0.64	0.61	0.65	0.15	0.00125	1.09
ATAD3C	A-C	0.87	0.68	-0.15	0.07	-0.43	0.24	0.00127	1.30
FBXL5	B-A	2.37	0.63	3.76	0.38	3.61	0.35	0.00136	1.39
FUCA1	C-A	0.03	0.04	0.66	0.15	0.84	0.13	0.00136	0.81
PSME2	C-A	-0.23	0.46	0.61	0.47	1.02	0.41	0.00147	1.25
PANK2	B-A	0.53	0.39	1.54	0.25	1.43	0.40	0.00159	1.01
CGB1	A-C	-1.07	0.43	-2.07	0.10	-2.44	0.13	0.00163	1.38
GCA	C-A	1.84	0.05	3.64	0.08	4.07	0.47	0.00167	2.24
TIA1	C-B	0.80	0.18	0.69	0.21	1.43	0.23	0.00168	0.74
LYAR	C-A	-0.89	0.32	-0.60	0.22	-0.08	0.23	0.00180	0.81
PCSK1N	A-B	-0.58	0.17	-1.70	0.25	-1.88	0.00	0.00184	1.12
SHC1	B-A	-0.70	0.00	0.27	0.04			0.00186	0.97
ARMC1	C-A	0.12	0.49	1.01	0.32	1.22	0.37	0.00186	1.09
MOB1A	C-A	1.39	0.43	2.06	0.19	2.43	0.20	0.00190	1.04
DNAJA1	C-A	-0.10	0.48	0.73	0.32	1.07	0.37	0.00192	1.18
AVP	A-B	1.01	0.19	0.42	0.03	0.51	0.15	0.00192	0.59
KLF2	B-A	4.67	0.39	6.21	0.36	5.80	0.55	0.00195	1.54
OAZ1	C-A	1.10	0.61	2.07	0.40	2.28	0.20	0.00197	1.17
MAFF	A-C	1.19	0.46	0.43	0.00	-0.11	0.16	0.00199	1.30
ARID4B	B-A	1.34	0.49	2.39	0.37	2.22	0.26	0.00207	1.05
C9orf78	B-A	1.38	0.29	2.19	0.42	1.98	0.23	0.00209	0.81
C19orf6	A-C	-0.66	0.06	-1.45	0.00	-1.54	0.22	0.00214	0.88
H3F3C	A-B	0.49	0.61	-0.88	0.39	-0.55	0.40	0.00214	1.37
TSPAN13	C-A	-1.14	0.09	-0.54	0.10	0.04	0.13	0.00219	1.18
TATDN2	B-A	0.96	0.16	1.81	0.15	1.09	0.03	0.00222	0.85
ARHGAP17	B-A	-0.02	0.23	1.93	0.14	0.28	0.17	0.00228	1.95
FCN1	C-A	3.09	0.48	3.94	0.25	4.12	0.39	0.00229	1.03
SLC38A5	A-B	1.99	0.69	0.70	0.34	0.90	0.23	0.00231	1.29
ARPC1A	C-A	-0.73	0.38	0.12	0.18	0.19	0.27	0.00238	0.92
ZNF383	A-B	3.15	0.60	1.94	0.17	1.97	0.40	0.00242	1.22
FBXL3	C-A	1.09	0.08	1.60	0.02	1.72	0.10	0.00244	0.63
SRSF1	C-A	0.17	0.44	1.12	0.59	1.26	0.27	0.00244	1.09
SOD2	C-A	1.79	0.49	2.72	0.14	3.26	0.88	0.00245	1.47

PCNP	C-A	0.13	0.70	1.30	0.33	1.39	0.24	0.00246	1.26
ID3	A-C	-2.10	0.48	-3.02	0.16	-3.14	0.10	0.00254	1.04
KIAA1467	A-C	-0.37	0.50	-1.39	0.29	-1.50	0.25	0.00255	1.14
NAA20	C-B	0.05	0.12	-0.60	0.12	0.13	0.24	0.00262	0.73
IRF7	C-A	1.31	0.38	2.19	0.25	2.71	0.32	0.00266	1.40
SEMA4D	B-A	1.96	0.32	3.11	0.56	2.48	0.36	0.00268	1.15
OAT	C-A	0.11	0.08	0.47	0.09	0.65	0.18	0.00275	0.54
CALM1	B-A	-0.17	0.38	1.16	0.43	0.42	0.51	0.00275	1.32
FGFR4	C-A	1.93	0.22	2.25	0.68	3.16	0.30	0.00275	1.23
NCAPH2	A-C	1.38	0.50	0.81	0.07	0.37	0.35	0.00277	1.01
PREP	C-B			1.53	0.12	1.90	0.07	0.00278	0.38
SERINC1	B-A	1.67	0.59	2.86	0.41	2.72	0.33	0.00286	1.19
CASP1	C-A	2.82	0.79	4.57	0.30	4.65	0.62	0.00288	1.83
NAGK	C-A	1.19	0.35	1.58	0.46	2.43	0.24	0.00289	1.23
IFITM1	C-A	0.17	0.64	1.34	0.49	1.83	0.87	0.00291	1.66
CD58	B-A	0.96	0.33	2.26	0.25	1.95	0.27	0.00293	1.30
LRP6	A-C	1.51	1.18	0.39	0.32	-0.82	0.66	0.00293	2.33
TMSB4X	C-A	0.67	0.74	1.89	0.21	2.05	0.55	0.00298	1.38
HSPA8	C-A	-2.63	0.59	-1.50	0.14	-1.37	0.13	0.00300	1.26
HBD	B-A	-1.46	1.04	3.02	0.69	2.07	3.18	0.00305	4.48
LILRB2	C-A	3.37	0.04	3.55	0.00	5.69	0.29	0.00309	2.32
UG0898H09	A-B	2.79	0.25	1.28	0.22	0.37	0.00	0.00312	1.52
ARPC5	C-A	1.06	1.11	2.77	0.32	2.83	0.25	0.00314	1.77
USP8	B-A	0.57	0.33	1.91	0.41	1.54	0.22	0.00317	1.34
GGT1	A-B	-1.47	0.34	-2.74	0.12			0.00318	1.27
GTF2A1	C-A	1.02	0.27	1.58	0.36	1.90	0.19	0.00320	0.87
KRTAP21-1	A-B	-0.64	0.31	-1.41	0.22	-1.01	0.30	0.00330	0.77
RAB10	C-B	1.60	0.46	1.60	0.37	2.58	0.18	0.00331	0.98
LOC286272	A-C	2.64	1.11	1.67	0.30	0.55	0.30	0.00332	2.09
TAF2	A-C	-1.41	0.70	-2.21	0.10	-2.67	0.23	0.00333	1.25
ITM2B	C-A	0.54	0.80	1.81	0.22	1.99	0.56	0.00337	1.45
TACC1	A-B	1.38	0.28	0.08	0.10			0.00351	1.31
CDK10	A-B	-0.11	0.29	-0.77	0.31	-0.67	0.26	0.00356	0.65
MTPN	B-A	0.46	0.71	1.68	0.22	1.54	0.23	0.00358	1.22
B2M	C-A	0.99	1.13	2.47	0.11	2.98	0.57	0.00377	1.99
LOC100506990	A-B	1.19	0.36	0.39	0.25	0.45	0.27	0.00378	0.80
LSM14A	B-A	0.53	0.27	1.11	0.02	1.11	0.14	0.00383	0.58
C21orf30	A-C	-2.41	0.62	-3.08	0.23	-3.61	0.39	0.00384	1.21
PDCD10	C-A	0.99	0.71	1.84	0.35	2.32	0.24	0.00386	1.33
LOC100287934	A-C	2.18	0.64	1.44	0.14	0.94	0.24	0.00394	1.24
JAK1	C-A	0.88	0.24	1.42	0.03	1.70	0.11	0.00403	0.82
ARPC3	B-A	0.20	0.45	1.14	0.22	0.98	0.40	0.00409	0.94
ANKRD13A	C-A	2.29	0.59	3.15	0.28	3.33	0.19	0.00417	1.04
XRCC1	B-A	-0.20	0.11	0.66	0.09	-0.18	0.10	0.00418	0.86
PSMB2	C-A	-1.11	0.26	-0.73	0.18	-0.64	0.11	0.00419	0.48
PDE4B	A-C	-0.28	0.25	-1.22	0.00	-1.59	0.05	0.00419	1.30

SP2	A-B	2.26	0.80	1.02	0.25	1.06	0.22	0.00423	1.23
ILF3	A-B	-2.16	0.31	-3.64	0.21	-3.39	0.05	0.00426	1.49
PKNOX2	A-C	2.62	0.54	1.95	0.53	1.51	0.20	0.00431	1.11
MAP3K4	A-C	-2.33	0.21	-3.32	0.26	-4.11	0.04	0.00437	1.78
TMCO1	C-B	-0.02	0.14	-0.25	0.14	0.20	0.08	0.00439	0.45
U2AF1L4	A-B	2.29	0.05	1.77	0.05	2.25	0.14	0.00441	0.52
SNX10	C-A	2.37	0.77	3.63	0.40	3.85	0.67	0.00445	1.49
FGR	C-A	1.20	0.04	2.66	0.00	2.49	0.08	0.00445	1.29
PYCARD	C-A	1.74	0.43	2.36	0.36	2.64	0.28	0.00446	0.91
SIGLEC11	A-B	0.42	0.24	-1.05	0.25			0.00447	1.47
POM121	A-C	0.01	0.23	-1.17	0.07	-1.48	0.36	0.00449	1.49
RBM5	B-A	2.24	0.47	3.11	0.24	2.99	0.08	0.00449	0.87
CHST15	B-A	2.64	0.47	3.82	0.22	3.45	0.51	0.00449	1.18
MICA	A-B	-1.96	0.22	-3.14	0.19	-3.53	0.00	0.00450	1.18
STAG2	B-A	0.63	0.47	1.65	0.36	1.35	0.24	0.00454	1.02
SNW1	B-A	0.70	0.44	1.57	0.43	1.52	0.34	0.00456	0.87
ZNF480	A-C	2.06	0.76	1.45	0.20	0.54	0.11	0.00467	1.52
GPR161	A-B	2.26	0.14	1.11	0.10			0.00477	1.15
TBK1	C-A	0.81	0.35	1.56	0.56	1.89	0.26	0.00477	1.09
HLA-H	C-A	0.54	0.76	1.58	0.26	1.88	0.47	0.00494	1.34
CKLF	B-A	-0.05	0.44	0.84	0.38	0.74	0.38	0.00494	0.88
FBXO34	B-A	0.90	0.27	2.05	0.11	0.92	0.09	0.00509	1.16
IL6ST	B-A	-1.10	0.24	0.06	0.39	-0.06	0.55	0.00511	1.16
ST7L	A-B	-1.40	0.08	-2.60	0.36	-3.08	0.00	0.00512	1.20
TMA7	C-A	0.39	0.43	0.94	0.12	1.11	0.20	0.00513	0.72
SORL1	B-A	1.87	0.52	3.03	0.41	2.81	0.21	0.00516	1.16
KRT15	A-C	3.18	0.84	2.44	0.14	1.69	0.20	0.00523	1.48
OCIAD1	C-A	1.04	0.57	1.79	0.24	1.97	0.14	0.00524	0.93
KIAA2013	B-A	0.15	0.08	1.11	0.21	1.09	0.06	0.00527	0.96
PEX19	A-C	0.82	1.06	-0.56	0.41	-0.88	0.18	0.00529	1.70
MTCP1	A-B	0.06	0.73	-1.40	0.29	-1.29	0.29	0.00537	1.46
RHOC	A-B	-1.20	0.38	-1.89	0.25	-1.81	0.25	0.00537	0.70
PDS5A	B-C	0.45	0.13	1.04	0.12	0.39	0.11	0.00550	0.65
SKAP2	B-A	1.86	0.40	2.93	0.47	2.81	0.40	0.00555	1.06
PRB1	B-A	1.98	0.25	3.10	0.29	2.28	0.55	0.00558	1.12
CHPT1	B-A	0.03	0.58	1.31	0.52	1.09	0.11	0.00560	1.28
RGS19	B-A	1.26	0.40	2.48	0.74	2.39	0.48	0.00560	1.22
ORAI1	A-C	0.98	0.30	0.66	0.25	0.27	0.30	0.00562	0.71
PPIB	A-C	-1.82	0.09			-2.32	0.21	0.00567	0.49
CDKN2B	A-C	0.64	1.05	-0.37	0.34	-1.43	0.72	0.00567	2.07
DHPS	A-C	1.08	0.40	0.65	0.41	0.22	0.24	0.00567	0.86
IDI1	C-B	0.75	0.44	-0.06	0.34	0.98	0.25	0.00567	1.04
HCN2	A-B	-0.65	0.12	-1.50	0.22	-1.17	0.23	0.00573	0.85
FKBP5	C-A	0.21	0.02	1.03	0.00	1.15	0.14	0.00574	0.94
ATP5H	B-A	-1.54	0.42	-0.72	0.53	-0.75	0.24	0.00574	0.82
CPVL	B-A	2.35	0.67	3.65	0.32	3.63	0.48	0.00574	1.30

CDRT1	A-C	2.07	1.30	1.07	0.32	-0.37	0.87	0.00581	2.44
ACTB	B-A	-1.69	0.64	-0.14	0.85	-0.80	0.40	0.00582	1.56
NCOA4	B-A	1.01	0.76	2.26	0.29	2.04	0.22	0.00584	1.24
RUVBL2	A-C	-0.41	0.40	-1.09	0.15	-1.11	0.36	0.00593	0.70
SELO	A-B	1.76	0.35	1.00	0.34	1.08	0.26	0.00594	0.75
CBWD3	C-A	0.68	0.47	1.38	0.44	1.61	0.22	0.00600	0.92
TMSB4X	C-A	-0.40	0.80	1.03	0.64	1.19	0.81	0.00603	1.59
SIDT2	B-A	1.39	0.22	2.38	0.39	2.29	0.29	0.00603	0.99
CERS2	B-A	0.65	0.44	1.76	0.43	1.30	0.23	0.00604	1.11
CFL1P1	A-C	-1.81	0.05	-3.03	0.00	-2.83	0.15	0.00604	1.02
TATDN3	C-A	0.60	0.06	0.82	0.00	1.27	0.11	0.00607	0.67
ECM1	A-B	0.44	0.27	-0.59	0.02			0.00611	1.03
B2M	C-A	0.78	1.19	2.13	0.11	2.77	0.53	0.00612	1.98
CSNK1A1	B-A	0.57	0.50	1.55	0.59	1.54	0.28	0.00617	0.99
ACTR2	B-A	0.53	0.65	1.74	0.50	1.64	0.43	0.00618	1.21
CDC42EP3	B-A	1.01	0.12	2.09	0.29	1.83	0.33	0.00620	1.08
SON	B-A	0.24	0.51	1.45	0.61	1.00	0.35	0.00623	1.22
DNAJA1	C-A	0.07	0.32	0.57	0.34	0.77	0.23	0.00625	0.70
CCL4	A-C	1.23	0.69	0.34	0.16	0.17	0.20	0.00629	1.06
DOM3Z	A-C	1.29	0.52	0.77	0.13	0.33	0.10	0.00636	0.97
DENR	C-B	1.27	0.37	0.97	0.27	1.85	0.25	0.00641	0.88
PGR	A-C	2.97	0.15	2.56	0.20	1.93	0.08	0.00643	1.04
NPC2	C-A	0.70	0.36	1.58	0.38	1.65	0.66	0.00649	0.95
BIRC2	C-A	1.34	0.59	2.13	0.13	2.28	0.33	0.00654	0.95
CYTH1	B-A	1.67	0.39	2.86	0.59	2.17	0.54	0.00656	1.19
FLJ31662	A-B	3.60	0.11	2.03	0.07	1.39	0.00	0.00668	1.57
NCF2	B-A	2.05	0.09	4.23	0.65	4.05	0.50	0.00669	2.18
RNASET2	B-A	1.44	0.31	2.20	0.36	1.87	0.30	0.00670	0.76
OPA3	A-C	-0.33	0.83	-1.18	0.40	-1.91	0.20	0.00673	1.58
C1QL1	A-C	-0.63	0.71	-1.39	0.23	-1.77	0.15	0.00674	1.14
ELAVL1	C-B	-0.12	0.05	-0.17	0.05	0.44	0.16	0.00682	0.61
DTX2	C-A	1.56	0.27	1.98	0.07	2.05	0.18	0.00691	0.49
GPBP1	C-A	1.25	0.58	1.98	0.37	2.31	0.36	0.00692	1.06
HNMT	C-A	0.72	0.09	1.91	0.20	2.15	0.44	0.00696	1.42
OS9	B-A	0.03	0.28	0.89	0.60	0.54	0.23	0.00712	0.86
EEF1A1	C-B	-1.98	0.47	-3.26	1.48	-1.12	0.22	0.00713	2.14
RPSAP58	C-A	-0.54	0.29	-0.39	0.48	0.29	0.39	0.00713	0.83
DERL2	C-B	1.35	0.19	0.74	0.00	1.44	0.17	0.00717	0.70
TMSB4XP2	B-A	-1.15	0.43	-0.25	0.35	-0.33	0.54	0.00728	0.90
OR10C1	A-C	2.91	0.55	2.20	0.47	1.63	0.33	0.00731	1.28
PLEKHA2	C-A	2.13	0.53	2.87	0.32	3.00	0.28	0.00733	0.88
SERPINE2	A-C	-2.22	0.43	-3.05	0.17	-3.24	0.71	0.00733	1.02
SNX14	C-A	1.71	0.56	2.58	0.28	2.60	0.38	0.00734	0.89
SDHB	C-A	-0.29	0.39	0.51	0.39	0.52	0.47	0.00740	0.80
ROMO1	A-B	-0.23	0.39	-0.94	0.15	-0.69	0.25	0.00744	0.71
APBB1	A-C	2.29	0.18	1.91	0.04	1.52	0.12	0.00745	0.78

ARHGAP35	A-C	2.27	1.07	0.86	0.51	0.56	0.48	0.00749	1.71
RPL13AP6	A-B	-2.96	0.45	-3.75	0.35	-3.68	0.33	0.00772	0.79
DOT1L	A-B	-1.14	0.06	-2.26	0.08	-2.40	0.00	0.00773	1.12
KYNU	C-A	0.62	0.55	1.49	0.29	1.62	0.53	0.00775	1.01
HLA-DQB1	B-A	4.35	0.43	5.50	0.18	5.19	0.42	0.00778	1.15
GALNT7	B-A	1.03	0.46	1.77	0.31	1.77	0.25	0.00802	0.74
NCF1	C-A	1.69	0.77	2.79	0.27	3.07	0.71	0.00806	1.38
SIGLEC8	A-C	-1.59	0.24	-2.78	0.30	-3.09	0.19	0.00810	1.50
NEK7	B-A	0.87	0.37	1.89	0.41	1.84	0.44	0.00837	1.02
FLOT2	B-A	0.43	0.04	0.89	0.03	0.71	0.05	0.00842	0.45
YWHAH	B-A	0.17	0.52	1.23	0.27	1.00	0.40	0.00878	1.06
JKAMP	C-A	1.56	0.60	1.76	0.22	2.57	0.17	0.00889	1.01
DMWD	A-C	1.57	0.45	0.77	0.16	0.74	0.11	0.00889	0.83
CACUL1	A-C	0.22	0.85	-0.48	0.29	-1.16	0.24	0.00894	1.38
C8orf44	A-C	2.55	0.79	1.48	0.20	1.26	0.30	0.00894	1.29
SEPT7	C-A	-1.36	0.23	-0.77	0.28	-0.69	0.14	0.00903	0.67
GOLGA6L2	A-C	1.59	0.73	1.01	0.24	0.40	0.26	0.00907	1.19
ZFAND3	C-A	0.36	0.10	1.01	0.00	0.97	0.08	0.00910	0.60
FAM45A	C-A	1.52	0.45	2.41	0.31	2.57	0.45	0.00912	1.05
ERGIC1	A-B	1.55	0.47	0.50	0.28	0.56	0.46	0.00912	1.05
DAD1	C-A	-0.44	0.66	0.21	0.51	0.73	0.23	0.00913	1.17
TMSB4X	C-A	0.02	0.92	1.42	0.48	1.64	0.85	0.00916	1.62
KIF2A	C-A	1.14	0.80	2.24	0.15	2.40	0.48	0.00917	1.26
UBB	C-B	-1.66	0.51	-1.91	1.50	-0.04	0.40	0.00925	1.87
AZIN1	C-B	0.07	0.00	-0.24	0.26	0.70	0.22	0.00931	0.94
LAMP2	C-A	-0.02	0.28	0.61	0.27	0.91	0.37	0.00938	0.94
TOMM40	A-B	-0.37	0.19	-0.95	0.14	-0.60	0.37	0.00940	0.58
GLYR1	C-A	0.50	0.49	1.32	0.29	1.35	0.28	0.00951	0.85
MANBAL	A-C	-0.04	0.37	0.09	0.00	-0.91	0.31	0.00954	0.87
BLVRA	C-A	0.70	0.56	1.52	0.28	1.59	0.39	0.00958	0.89
GNAI2	C-A	2.03	0.60	2.90	0.21	2.93	0.38	0.00959	0.90
BNIP2	B-A	2.41	0.50	3.52	0.48	3.31	0.27	0.00962	1.11
EDEM2	B-A	1.32	0.39	1.97	0.25	1.91	0.23	0.00963	0.65
RFX5	B-A	1.57	0.40	2.41	0.45	2.13	0.20	0.00968	0.84
PXN	B-A	1.02	0.26	1.98	0.64	1.53	0.45	0.00980	0.96
IFI16	C-B	-0.04	0.26	-0.23	0.02	0.47	0.21	0.00980	0.70
SIRPB1	C-B	-1.09	0.40	-1.91	0.80	-0.57	0.37	0.01000	1.33

**ET**		**A**		**B**		**C**		**p value**	**Max. Mean Differ**
***JAK2*V617F**		**Control**		**No**		**heterozygote**			
**Gene**	**BG**	**Mean**	**SD**	**Mean**	**SD**	**Mean**	**SD**		
SOD2	B-A	1.79	0.49	3.35	0.22	3.32	0.52	0.0001	1.57
OAZ1	C-A	1.10	0.61	2.39	0.33	2.45	0.24	0.0001	1.35
CDC42EP3	C-A	1.01	0.12	2.14	0.00	2.29	0.17	0.0002	1.28
OAT	C-A	0.11	0.08	0.49	0.00	0.59	0.07	0.0002	0.48
CIB1	B-A	1.12	0.25	2.58	0.43	1.44	0.24	0.0003	1.46
BRI3	C-A	-0.06	0.11	0.30	0.39	0.98	0.41	0.0004	1.04

LCK	C-A	0.65	0.44	1.75	0.12	1.80	0.36	0.0004	1.15
TMSB4X	C-A	0.67	0.74	2.02	0.23	2.29	0.51	0.0004	1.62
TMSB4X	C-A	-0.40	0.80	1.02	0.43	1.36	0.55	0.0006	1.76
ACTB	C-A	-1.69	0.64	-0.58	0.43	0.29	0.93	0.0006	1.99
PF4V1	B-A	-0.26	0.25	2.24	0.13	1.32	0.37	0.0006	2.50
CEP89	A-B	-0.02	0.38	-1.63	0.20	-1.08	0.31	0.0009	1.61
IFITM1	C-A	0.17	0.64	1.20	0.17	1.30	0.21	0.0009	1.13
RAB8B	C-A	1.93	0.38	3.08	0.00	3.05	0.40	0.0011	1.12
TMEM60	C-A	-0.02	0.18	0.23	0.00	0.63	0.15	0.0011	0.65
REEP5	C-A	0.64	0.26	1.35	0.03	1.59	0.30	0.0012	0.95
TMSB4XP2	B-A	-1.15	0.43	-0.04	0.44	-0.37	0.40	0.0015	1.11
CSF3R	C-A	2.83	0.20	2.99	0.37	3.82	0.59	0.0015	0.99
NFXL1	A-B	1.21	0.03	0.52	0.07	0.74	0.06	0.0016	0.69
TGFBI	C-A	-0.31	0.34	0.30	0.40	0.65	0.39	0.0017	0.96
TMSB4X	C-A	0.02	0.92	1.28	0.45	1.74	0.54	0.0021	1.72
SLC20A1	C-A	-0.06	0.17	0.32	0.32	0.79	0.36	0.0026	0.84
LOC124685	B-A	-0.67	0.24	0.38	0.54	-0.16	0.47	0.0026	1.06
SULF2	C-A	-0.81	0.30	-0.37	0.05	0.19	0.42	0.0029	1.00
DPF3	A-B	-0.84	0.10	-2.24	0.37	-1.57	0.22	0.0029	1.40
NCF1	C-A	1.69	0.77	2.01	0.71	3.35	0.63	0.0030	1.66
CKS2	A-C	-0.83	0.55	-1.73	0.21	-1.90	0.37	0.0030	1.06
PYCARD	C-A	1.74	0.43	2.26	0.36	2.94	0.54	0.0031	1.20
SLC27A3	C-A	1.35	0.54	1.99	0.52	2.77	0.59	0.0033	1.42
GPX1	B-A	1.38	0.46	2.72	0.70	2.35	0.51	0.0036	1.34
FCN1	C-A	3.09	0.48	3.55	0.73	4.45	0.55	0.0038	1.36
C12orf35	C-A	0.49	0.18	0.94	0.11	1.68	0.38	0.0038	1.19
LMBRD1	C-A	1.62	0.19	2.07	0.00	2.40	0.40	0.0039	0.78
RPL13AP6	A-C	-2.96	0.45	-3.65	0.51	-4.00	0.47	0.0039	1.04
BBC3	C-A	0.12	0.14	0.49	0.15	1.13	0.26	0.0041	1.01
ZFAND5	C-A	0.26	0.22	0.43	0.00	1.38	0.16	0.0044	1.12
IRF7	C-A	1.31	0.38	2.24	0.00	2.61	0.50	0.0045	1.29
EIF4H	A-C	-0.06	0.34	-0.54	0.23	-0.62	0.15	0.0045	0.56
MFSD1	B-A	1.52	0.49	2.91	0.74	2.59	0.60	0.0046	1.39
ST7L	A-B	-1.40	0.08	-3.09	0.22	-2.48	0.36	0.0048	1.68
KIAA0930	C-A	1.37	0.46	1.75	0.52	2.63	0.69	0.0052	1.26
CNIH4	C-A	-0.90	0.33	-0.37	0.07	-0.27	0.28	0.0052	0.63
DTX2	C-A	1.56	0.27	2.33	0.00	2.32	0.46	0.0053	0.77
CHMP5	C-A	0.44	0.68	1.20	0.41	1.67	0.45	0.0054	1.22
CFL1P1	A-C	-1.81	0.05	-2.79	0.00	-2.95	0.06	0.0055	1.14
PANK2	C-A	0.53	0.39	0.82	0.25	1.41	0.37	0.0055	0.88
ITM2B	B-A	0.54	0.80	1.79	0.22	1.69	0.50	0.0056	1.26
WAC	C-A	-0.20	0.41	0.30	0.73	0.94	0.43	0.0058	1.13
61E3.4	A-B	-1.04	0.33	-1.78	0.29	-1.41	0.28	0.0059	0.74
FTL	B-A	-0.73	0.41	0.00	0.44	-0.02	0.27	0.0059	0.73
CTNND1	A-B	-0.19	0.41	-1.64	0.21	-0.72	0.38	0.0060	1.45
HIST2H3C	B-A	-1.58	0.55	-0.72	0.11	-0.78	0.39	0.0063	0.86

NOP58	B-C	0.95	0.00	0.33	0.05	-0.19	0.07	0.0064	0.52
CST3	C-A	0.68	0.43	1.17	0.35	1.54	0.35	0.0065	0.86
MAST3	A-C	2.79	0.00	0.67	0.00	1.43	0.11	0.0065	1.36
PSMB9	C-A	1.49	0.44	2.30	0.23	2.64	0.57	0.0066	1.16
PRR13	C-A	0.28	0.43	0.78	0.26	0.96	0.20	0.0068	0.69
TATDN3	C-A	0.60	0.06	0.96	0.00	1.00	0.03	0.0068	0.40
S100A6	C-A	0.46	0.53	0.96	0.77	1.61	0.31	0.0068	1.14
P4HTM	C-A	0.80	0.07	1.04	0.00	1.90	0.06	0.0069	1.10
HN1	B-A	-0.83	0.19	-0.38	0.35	-0.42	0.18	0.0069	0.46
TNFRSF1A	C-B	0.58	0.67	0.50	0.59	1.76	0.50	0.0070	1.26
LOC401188	A-C	0.13	0.27			-1.14	0.16	0.0070	1.28
CDK4	A-C	-0.95	0.30	-1.66	0.24	-1.75	0.26	0.0076	0.80
CALM1	C-A	-0.17	0.38	0.42	0.31	0.64	0.38	0.0076	0.81
CCND3	B-A	0.21	0.22	1.11	0.49	0.94	0.40	0.0076	0.90
PPP2R5C	C-A	-0.60	0.16	-0.39	0.05	-0.12	0.24	0.0078	0.48
HLA-G	B-A	1.16	0.37	1.92	0.14	1.78	0.48	0.0078	0.76
FBXL5	C-A	2.37	0.63	2.41	0.51	3.48	0.48	0.0080	1.11
S100A4	C-A	2.34	0.70	3.08	0.58	3.55	0.41	0.0081	1.22
MTCH1	A-C	0.97	0.36	0.83	0.25	0.31	0.08	0.0082	0.66
HSPD1	A-C	-2.00	0.43	-2.62	0.40	-2.65	0.08	0.0083	0.64
PXN	C-A	1.02	0.26	1.59	0.11	1.93	0.62	0.0084	0.91
AP1AR	C-B	1.33	0.13	0.70	0.47	1.73	0.18	0.0084	1.03
HSPE1	A-B	-2.17	0.34	-2.80	0.38	-2.72	0.26	0.0089	0.63
CSNK1A1	C-A	0.57	0.50	1.20	0.19	1.49	0.48	0.0089	0.92
USP11	A-C	-0.49	0.40	-1.17	0.08	-1.19	0.41	0.0091	0.70
SNRPD3	A-B	-0.49	0.18	-0.91	0.15	-0.88	0.10	0.0092	0.42
COTL1	B-A	0.85	0.47	1.65	0.41	1.60	0.33	0.0092	0.80
FCN2	A-C	2.10	0.25			0.67	0.09	0.0094	1.43
RNF144A	A-C	1.54	0.59	1.03	0.20	0.65	0.25	0.0096	0.89
TSPO	C-A	1.12	0.44	2.31	0.00	1.79	0.22	0.0100	0.67
**PMF**		**A**		**B**		**C**		**p value**	**Max. Mean Differ**
***JAK2*V617F**		**Control**		**No**		**heterozygote**			
**Gene**	**BG**	**Mean**	**SD**	**Mean**	**SD**	**Mean**	**SD**		
DBI	A-C	-1.28	0.06	-1.36	0.09	-2.01	0.01	0.0001	0.73
LAMP1	C-A	-0.27	0.38	0.86	0.37	1.06	0.03	0.0023	1.33
RAP1B	B-A	0.67	0.52	2.64	0.14	1.86	0.51	0.0029	1.97
CNTROB	A-B	3.08	0.32	1.90	0.29	2.52	0.13	0.0032	1.18
STAT5A	A-B	1.39	0.25	0.53	0.25	1.93	0.00	0.0046	0.85
POLR2E	B-A	0.10	0.52	1.84	0.11	0.89	0.37	0.0046	1.74
ARL5A	B-C	1.26	0.17	1.65	0.10	0.73	0.16	0.0049	0.92
TOB1	C-A	1.88	0.79	3.29	0.00	4.15	0.23	0.0053	2.27
TPP2	B-C	1.52	0.25	2.48	0.11	1.27	0.31	0.0055	1.22
ARIH1	B-C	1.02	0.09	1.43	0.11	0.43	0.13	0.0055	1.00
EGFL8	A-C	1.27	0.18	0.16	0.00	0.76	0.03	0.0060	0.52
HIPK4	A-B	0.38	0.28	-0.46	0.18	-0.14	0.00	0.0065	0.84
HMGN2	B-A	-1.97	0.28	-0.97	0.43	-1.22	0.25	0.0081	1.00

PARP9	C-A	0.62	0.21	0.94	0.29	2.01	0.24	0.0083	1.38
CAPNS1	B-C	-0.99	0.11	-0.77	0.04	-1.40	0.10	0.0085	0.64
IL23A	A-C	-1.18	0.16	-1.26	0.18	-1.71	0.02	0.0090	0.52
RPSAP58	B-A	-0.54	0.29	0.38	0.41	0.18	0.24	0.0091	0.92
LOC124685	B-A	-0.67	0.24	0.16	0.24	-0.35	0.30	0.0106	0.83
SOD2	C-A	1.79	0.49	3.48	0.00	3.40	0.93	0.0117	1.62
UBB	B-A	-1.66	0.51	-0.38	0.04	-0.73	0.25	0.0122	1.28
ZNF728	C-A	-1.10	0.18	-0.50	0.36	-0.42	0.27	0.0124	0.68
CCND3	B-A	0.21	0.22	1.21	0.40	0.76	0.21	0.0136	1.01
HMG1L10	B-A	-0.76	0.42	0.49	0.46	-0.19	0.16	0.0148	1.25
SERHL2	A-B	-0.17	0.41	-1.37	0.28	-0.38	0.00	0.0154	1.20
NSUN5	A-B	0.27	0.16	-0.19	0.16	0.32	0.00	0.0154	0.47
HLA-E	B-A	1.76	0.55	3.31	0.00	2.60	0.29	0.0159	1.55
AVP	C-A	1.01	0.19	1.58	0.00	1.83	0.32	0.0174	0.82
GADD45B	A-C	1.26	0.43	1.12	0.00	-0.27	1.11	0.0177	1.53
TNFAIP3	A-C	2.69	0.71	2.54	0.00	1.00	0.53	0.0180	1.69
RFWD2	B-A	1.47	0.16	2.08	0.09	1.63	0.16	0.0181	0.61
MAFF	A-C	1.19	0.46	0.38	0.00	0.08	0.31	0.0200	1.12
HN1	B-A	-0.83	0.19	-0.18	0.16	-0.58	0.35	0.0207	0.66
EIF4A1	B-C	0.17	0.42	0.72	0.16	-0.73	0.30	0.0211	1.46
HSP90B1	B-A	-1.44	0.48	-0.13	0.11	-0.99	0.30	0.0216	1.31
ZNF708	B-A	-0.51	0.37	0.35	0.39	0.26	0.22	0.0219	0.86
VPS8	B-C	-0.16	0.16	0.57	0.09	-0.47	0.40	0.0227	1.04
C1orf63	B-C	1.57	0.05	1.94	0.22	1.33	0.03	0.0235	0.61
hsa-mir-425	C-B	0.61	0.60	0.26	0.35	2.10	0.32	0.0241	1.83
DAD1	B-A	-0.44	0.66	1.00	0.48	0.80	0.23	0.0242	1.44
YBX1	B-A	-3.27	0.31	-2.35	0.50	-2.69	0.06	0.0244	0.92
YWHAH	C-A	0.17	0.52	1.32	0.25	1.52	0.38	0.0251	1.34
SF3B3	A-B	-0.86	0.32	-1.63	0.03	-0.76	0.00	0.0253	0.77
TCEAL4	A-B	0.19	0.07	-0.28	0.19	0.15	0.04	0.0257	0.47
LGALS9	A-B	1.10	0.18	0.30	0.35	0.48	0.45	0.0258	0.79
LOC220906	B-A	0.92	0.33	1.83	0.26	1.54	0.49	0.0262	0.91
NCKAP5L	A-B	1.88	0.48	0.60	0.47	1.19	0.17	0.0263	1.28
CHMP5	C-A	0.44	0.68	1.73	0.18	1.73	0.22	0.0274	1.29
ANKRD62	B-A	-2.10	0.30	-1.36	0.19	-1.65	0.25	0.0277	0.74
PTGES3	B-A	-0.82	0.44	0.54	0.55	-0.01	0.40	0.0288	1.36
STOM	C-A	0.65	0.47	1.97	0.49	2.07	0.38	0.0290	1.43
OST4	C-A	-0.74	0.58	0.84	0.00	0.58	0.54	0.0298	1.32
FCHO1	A-B	1.97	0.48	0.72	0.35	1.25	0.55	0.0304	1.25
DGCR5	A-C	1.20	0.33	0.27	0.00	0.46	0.35	0.0310	0.74
ATP5H	B-A	-1.54	0.42	-0.61	0.00	-0.90	0.35	0.0316	0.93
HIST2H3D	B-A	-1.77	0.64	-0.46	0.41	-0.63	0.32	0.0318	1.31
GAPDH	B-A	-2.49	0.38	-1.66	0.13	-2.01	0.08	0.0319	0.83
NUPL1	B-A	0.03	0.18	0.59	0.07	0.16	0.03	0.0328	0.56
IGJ	A-C	-1.28	0.50	-2.16	0.64	-2.53	0.03	0.0340	1.25
MRPS21	A-B	-0.52	0.26	-1.08	0.09	-0.92	0.18	0.0361	0.56

PGRMC1	C-A	0.05	0.57	1.24	0.34	2.11	0.86	0.0374	2.06
HMGN2	B-A	-0.84	0.51	0.39	0.11	0.04	0.73	0.0376	1.23
WAC	B-A	-0.20	0.41	0.77	0.19	-0.11	0.02	0.0378	0.97
CCL3	A-C	2.45	0.40	1.46	0.09	1.46	0.75	0.0379	0.99
SEPT7	B-A	-1.36	0.23	-0.37	0.41	-0.99	0.24	0.0393	0.98
ZNF383	A-C	3.15	0.60	1.72	0.00	1.87	0.57	0.0400	1.29
SP2	A-B	2.26	0.80	0.53	0.28	1.26	0.47	0.0400	1.73
NDUFC2	B-A	-1.39	0.49	-0.28	0.26	-0.56	0.57	0.0410	1.11
KRT14	A-B	2.35	0.58	0.88	0.46	1.64	0.66	0.0410	1.47
PPTC7	A-C	1.70	0.32	1.56	0.54	0.68	0.08	0.0411	1.01
ZNF208	B-A	-1.69	0.24	-0.86	0.78	-1.04	0.04	0.0416	0.83
hsa-mir-373	C-B	1.19	0.81	0.93	0.30	3.03	0.67	0.0420	2.10
MAD2L1BP	B-A	0.62	0.31	1.43	0.34	0.88	0.27	0.0422	0.81
HIGD1A	B-A	-0.69	0.48	0.46	0.12	-0.16	0.04	0.0423	1.15
ZNF34	A-C	0.24	0.15	0.54	0.00	-0.10	0.02	0.0424	0.33
ARF1	B-A	0.57	0.37	1.37	0.00	0.99	0.07	0.0426	0.80
ACSL4	A-B	2.37	0.99	0.12	0.51	1.98	0.13	0.0429	2.25
LPCAT4	A-C	0.95	0.19	-0.14	0.00	0.54	0.16	0.0431	0.41
TGIF2-C20ORF24	B-A	-0.62	0.26	0.23	0.17	-0.29	0.17	0.0435	0.84
HMGN2	B-A	-1.43	0.35	-0.54	0.55	-0.67	0.12	0.0439	0.89
LGALS1	B-C	-1.41	0.40	-1.15	0.53	-2.42	0.39	0.0449	1.27
UNC119	A-C	1.34	0.25	1.60	0.00	0.83	0.18	0.0456	0.51
NR2F2	A-B	0.45	0.64	-0.96	0.18	-0.64	0.88	0.0465	1.41
AIF1	B-A	2.98	0.48	3.96	0.23	3.65	0.09	0.0465	0.98
C8orf44	A-B	2.55	0.79	0.74	0.27	2.05	0.38	0.0466	1.81
SEC24C	A-B	0.60	0.25	-0.61	0.94	0.34	0.49	0.0469	1.21
SORL1	B-A	1.87	0.52	3.01	0.09	2.35	0.00	0.0471	1.14
CD63	B-A	-1.59	0.22	-1.01	0.07	-1.55	0.34	0.0471	0.59
DBI	A-C	-1.09	0.10	-1.14	0.12	-1.61	0.03	0.0475	0.52
FMNL1	A-C	1.47	0.25	1.94	0.00	0.99	0.07	0.0478	0.47
ECM1	A-C	0.44	0.27			-0.49	0.53	0.0480	0.94
ID3	A-C	-2.10	0.48	-3.05	0.49	-3.12	0.16	0.0482	1.01
SEPT2	B-A	-0.13	0.29	0.74	0.44	0.14	0.33	0.0483	0.87
SUMO2	B-A	-1.82	0.40	-0.97	0.35	-1.22	0.22	0.0490	0.84
MTCH1	B-C	0.97	0.36	1.07	0.39	0.10	0.19	0.0498	0.98

DBI	A-C	-1.28	0.06	-1.36	0.09	-2.01	0.01	0.0001	0.73
LAMP1	C-A	-0.27	0.38	0.86	0.37	1.06	0.03	0.0023	1.33
RAP1B	B-A	0.67	0.52	2.64	0.14	1.86	0.51	0.0029	1.97
CNTROB	A-B	3.08	0.32	1.90	0.29	2.52	0.13	0.0032	1.18
STAT5A	A-B	1.39	0.25	0.53	0.25	1.93	0.00	0.0046	0.85
POLR2E	B-A	0.10	0.52	1.84	0.11	0.89	0.37	0.0046	1.74
ARL5A	B-C	1.26	0.17	1.65	0.10	0.73	0.16	0.0049	0.92
TOB1	C-A	1.88	0.79	3.29	0.00	4.15	0.23	0.0053	2.27
TPP2	B-C	1.52	0.25	2.48	0.11	1.27	0.31	0.0055	1.22
ARIH1	B-C	1.02	0.09	1.43	0.11	0.43	0.13	0.0055	1.00
EGFL8	A-C	1.27	0.18	0.16	0.00	0.76	0.03	0.0060	0.52
HIPK4	A-B	0.38	0.28	-0.46	0.18	-0.14	0.00	0.0065	0.84
HMGN2	B-A	-1.97	0.28	-0.97	0.43	-1.22	0.25	0.0081	1.00
PARP9	C-A	0.62	0.21	0.94	0.29	2.01	0.24	0.0083	1.38
CAPNS1	B-C	-0.99	0.11	-0.77	0.04	-1.40	0.10	0.0085	0.64
IL23A	A-C	-1.18	0.16	-1.26	0.18	-1.71	0.02	0.0090	0.52
RPSAP58	B-A	-0.54	0.29	0.38	0.41	0.18	0.24	0.0091	0.92
LOC124685	B-A	-0.67	0.24	0.16	0.24	-0.35	0.30	0.0106	0.83
SOD2	C-A	1.79	0.49	3.48	0.00	3.40	0.93	0.0117	1.62
UBB	B-A	-1.66	0.51	-0.38	0.04	-0.73	0.25	0.0122	1.28
ZNF728	C-A	-1.10	0.18	-0.50	0.36	-0.42	0.27	0.0124	0.68
CCND3	B-A	0.21	0.22	1.21	0.40	0.76	0.21	0.0136	1.01
HMG1L10	B-A	-0.76	0.42	0.49	0.46	-0.19	0.16	0.0148	1.25
SERHL2	A-B	-0.17	0.41	-1.37	0.28	-0.38	0.00	0.0154	1.20
NSUN5	A-B	0.27	0.16	-0.19	0.16	0.32	0.00	0.0154	0.47
HLA-E	B-A	1.76	0.55	3.31	0.00	2.60	0.29	0.0159	1.55
AVP	C-A	1.01	0.19	1.58	0.00	1.83	0.32	0.0174	0.82
GADD45B	A-C	1.26	0.43	1.12	0.00	-0.27	1.11	0.0177	1.53
TNFAIP3	A-C	2.69	0.71	2.54	0.00	1.00	0.53	0.0180	1.69
RFWD2	B-A	1.47	0.16	2.08	0.09	1.63	0.16	0.0181	0.61
MAFF	A-C	1.19	0.46	0.38	0.00	0.08	0.31	0.0200	1.12
HN1	B-A	-0.83	0.19	-0.18	0.16	-0.58	0.35	0.0207	0.66
EIF4A1	B-C	0.17	0.42	0.72	0.16	-0.73	0.30	0.0211	1.46
HSP90B1	B-A	-1.44	0.48	-0.13	0.11	-0.99	0.30	0.0216	1.31
ZNF708	B-A	-0.51	0.37	0.35	0.39	0.26	0.22	0.0219	0.86
VPS8	B-C	-0.16	0.16	0.57	0.09	-0.47	0.40	0.0227	1.04
C1orf63	B-C	1.57	0.05	1.94	0.22	1.33	0.03	0.0235	0.61
hsa-mir-425	C-B	0.61	0.60	0.26	0.35	2.10	0.32	0.0241	1.83
DAD1	B-A	-0.44	0.66	1.00	0.48	0.80	0.23	0.0242	1.44
YBX1	B-A	-3.27	0.31	-2.35	0.50	-2.69	0.06	0.0244	0.92
YWHAH	C-A	0.17	0.52	1.32	0.25	1.52	0.38	0.0251	1.34
SF3B3	A-B	-0.86	0.32	-1.63	0.03	-0.76	0.00	0.0253	0.77
TCEAL4	A-B	0.19	0.07	-0.28	0.19	0.15	0.04	0.0257	0.47
LGALS9	A-B	1.10	0.18	0.30	0.35	0.48	0.45	0.0258	0.79
LOC220906	B-A	0.92	0.33	1.83	0.26	1.54	0.49	0.0262	0.91
NCKAP5L	A-B	1.88	0.48	0.60	0.47	1.19	0.17	0.0263	1.28
CHMP5	C-A	0.44	0.68	1.73	0.18	1.73	0.22	0.0274	1.29
ANKRD62	B-A	-2.10	0.30	-1.36	0.19	-1.65	0.25	0.0277	0.74
PTGES3	B-A	-0.82	0.44	0.54	0.55	-0.01	0.40	0.0288	1.36

STOM	C-A	0.65	0.47	1.97	0.49	2.07	0.38	0.0290	1.43
OST4	C-A	-0.74	0.58	0.84	0.00	0.58	0.54	0.0298	1.32
FCHO1	A-B	1.97	0.48	0.72	0.35	1.25	0.55	0.0304	1.25
DGCR5	A-C	1.20	0.33	0.27	0.00	0.46	0.35	0.0310	0.74
ATP5H	B-A	-1.54	0.42	-0.61	0.00	-0.90	0.35	0.0316	0.93
HIST2H3D	B-A	-1.77	0.64	-0.46	0.41	-0.63	0.32	0.0318	1.31
GAPDH	B-A	-2.49	0.38	-1.66	0.13	-2.01	0.08	0.0319	0.83
NUPL1	B-A	0.03	0.18	0.59	0.07	0.16	0.03	0.0328	0.56
IGJ	A-C	-1.28	0.50	-2.16	0.64	-2.53	0.03	0.0340	1.25
MRPS21	A-B	-0.52	0.26	-1.08	0.09	-0.92	0.18	0.0361	0.56
PGRMC1	C-A	0.05	0.57	1.24	0.34	2.11	0.86	0.0374	2.06
HMGN2	B-A	-0.84	0.51	0.39	0.11	0.04	0.73	0.0376	1.23
WAC	B-A	-0.20	0.41	0.77	0.19	-0.11	0.02	0.0378	0.97
CCL3	A-C	2.45	0.40	1.46	0.09	1.46	0.75	0.0379	0.99
SEPT7	B-A	-1.36	0.23	-0.37	0.41	-0.99	0.24	0.0393	0.98
ZNF383	A-C	3.15	0.60	1.72	0.00	1.87	0.57	0.0400	1.29
SP2	A-B	2.26	0.80	0.53	0.28	1.26	0.47	0.0400	1.73
NDUFC2	B-A	-1.39	0.49	-0.28	0.26	-0.56	0.57	0.0410	1.11
KRT14	A-B	2.35	0.58	0.88	0.46	1.64	0.66	0.0410	1.47
PPTC7	A-C	1.70	0.32	1.56	0.54	0.68	0.08	0.0411	1.01
ZNF208	B-A	-1.69	0.24	-0.86	0.78	-1.04	0.04	0.0416	0.83
hsa-mir-373	C-B	1.19	0.81	0.93	0.30	3.03	0.67	0.0420	2.10
MAD2L1BP	B-A	0.62	0.31	1.43	0.34	0.88	0.27	0.0422	0.81
HIGD1A	B-A	-0.69	0.48	0.46	0.12	-0.16	0.04	0.0423	1.15
ZNF34	A-C	0.24	0.15	0.54	0.00	-0.10	0.02	0.0424	0.33
ARF1	B-A	0.57	0.37	1.37	0.00	0.99	0.07	0.0426	0.80
ACSL4	A-B	2.37	0.99	0.12	0.51	1.98	0.13	0.0429	2.25
LPCAT4	A-C	0.95	0.19	-0.14	0.00	0.54	0.16	0.0431	0.41
TGIF2-C20ORF24	B-A	-0.62	0.26	0.23	0.17	-0.29	0.17	0.0435	0.84
HMGN2	B-A	-1.43	0.35	-0.54	0.55	-0.67	0.12	0.0439	0.89
LGALS1	B-C	-1.41	0.40	-1.15	0.53	-2.42	0.39	0.0449	1.27
UNC119	A-C	1.34	0.25	1.60	0.00	0.83	0.18	0.0456	0.51
NR2F2	A-B	0.45	0.64	-0.96	0.18	-0.64	0.88	0.0465	1.41
AIF1	B-A	2.98	0.48	3.96	0.23	3.65	0.09	0.0465	0.98
C8orf44	A-B	2.55	0.79	0.74	0.27	2.05	0.38	0.0466	1.81
SEC24C	A-B	0.60	0.25	-0.61	0.94	0.34	0.49	0.0469	1.21
SORL1	B-A	1.87	0.52	3.01	0.09	2.35	0.00	0.0471	1.14
CD63	B-A	-1.59	0.22	-1.01	0.07	-1.55	0.34	0.0471	0.59
DBI	A-C	-1.09	0.10	-1.14	0.12	-1.61	0.03	0.0475	0.52
FMNL1	A-C	1.47	0.25	1.94	0.00	0.99	0.07	0.0478	0.47
ECM1	A-C	0.44	0.27			-0.49	0.53	0.0480	0.94
ID3	A-C	-2.10	0.48	-3.05	0.49	-3.12	0.16	0.0482	1.01
SEPT2	B-A	-0.13	0.29	0.74	0.44	0.14	0.33	0.0483	0.87
SUMO2	B-A	-1.82	0.40	-0.97	0.35	-1.22	0.22	0.0490	0.84
MTCH1	B-C	0.97	0.36	1.07	0.39	0.10	0.19	0.0498	0.98
